# Unveiling Pharmacological Responses and Potential Targets Insights of Identified Bioactive Constituents of *Cuscuta reflexa* Roxb. Leaves through In Vivo and In Silico Approaches

**DOI:** 10.3390/ph13030050

**Published:** 2020-03-20

**Authors:** Md. Adnan, Md. Nazim Uddin Chy, A.T.M. Mostafa Kamal, Md. Riad Chowdhury, Md. Shariful Islam, Md. Amzad Hossain, Abu Montakim Tareq, Md. Imam Hossain Bhuiyan, Md Nasim Uddin, Afroza Tahamina, Md Obyedul Kalam Azad, Young Seok Lim, Dong Ha Cho

**Affiliations:** 1 Department of Bio-Health Technology, Kangwon National University, Chuncheon 24341, Korea; mdadnan1991.pharma@gmail.com (M.A.); azadokalam@gmail.com (M.O.K.A.); 2 Department of Pharmacy, International Islamic University Chittagong, Chittagong 4318, Bangladesh; nazim107282@gmail.com (M.N.U.C.); riadchy01@gmail.com (M.R.C.); shariful.islam02@northsouth.edu (M.S.I.); ctg.amzad.edu@gmail.com (M.A.H.); montakim0.abu@gmail.com (A.M.T.); imamhossain037@gmail.com (M.I.H.B.); mdnasimuddin2018@gmail.com (M.N.U.); 3 Drug Discovery, GUSTO A Research Group, Chittagong 4000, Bangladesh; 4 Beijing Advanced Innovation Center for Food Nutrition and Human Health, Beijing Technology and Business University, Beijing 100048, China; tahaminasompurna@gmail.com

**Keywords:** *Cuscuta reflexa*, anxiolytic, antidepressant, anti-nociceptive, antidiarrheal, molecular docking, ADME/T, admetSAR

## Abstract

*Cuscuta reflexa* Roxb. is traditionally used by the indigenous communities of Bangladesh to treat different diseases, such as pain, edema, tumor, jaundice, and skin infections. This study tested neuro-pharmacological, anti-nociceptive, and antidiarrheal activities by in vivo and in silico experiments for the metabolites extracted (methanol) from the leaves of *Cuscuta reflexa* (MECR). During the anxiolytic evaluation analyzed by elevated plus maze and hole board tests, MECR (200 and 400 mg/kg) exhibited a significant dose-dependent reduction of anxiety-like behavior in mice. Similarly, mice treated with MECR demonstrated a dose-dependent decrease in the time of immobility in both forced swimming and tail suspension tests. In addition, anti-nociceptive activity was assessed by the chemical-induced (acetic acid and formalin) pain models. In both cases, 400 mg/kg was found to be most effective and significantly (*p* < 0.001) inhibited acetic acid stimulated writhing and formalin-induced licking (pain response) in mice. Furthermore, antidiarrheal efficacy determined by the castor-oil induced diarrheal model manifested an evident inhibition of diarrheal stool frequency. In parallel, previously isolated bioactive compounds were documented based on the biological activities and subjected to in silico studies to correlate with the current pharmacological outcomes. The selected isolated compounds (15) displayed favorable binding affinities to potassium channels, human serotonin receptor, COX-1, COX-2, M3 muscarinic acetylcholine receptor, and 5-HT3 receptor proteins. Additionally, the ADME/T and toxicological properties were justified to unveil their drug-like properties and toxicity level. Overall, *Cuscuta reflexa* is bioactive and could be a potential source for the development of alternative medicine.

## 1. Introduction

Traditional medicine is the most available and affordable source of treatment for many diseases [1]. Since primitive times, several activities of medicinal plants have been discovered in practices of traditional medicine [2]. These activities are the consequence of various bioactive compounds that provide adequate defensive advantages to the plant [3]. Different plants possess different phytochemicals, leading to diversity in the activity of each plant. These phytochemicals can be isolated and used in the development of novel drugs. However, most of the diseases are currently being treated by using synthetic medicines, even though they hold numerous side effects [4]. Two such diseases are anxiety and depression, in which 3.8% and 3.4% of the global population respectively are suffering from [5]. Although some chemical drugs are available for treatment purposes, due to their side effects, many patients prefer herbal medicine [6].

Similarly, most of the analgesic drugs such as COX-2 inhibitors and opioids exhibit some unwanted adverse effects, including liver failure, GIT disorder, kidney dysfunction, and neutropenia [7,8]. Thus, medicinal plants can be an essential source for the development of analgesic drugs as they have wide traditional popularity [9]. Furthermore, diarrhea is another condition which is found to be the second leading cause of death of children and infant [10]. Due to the widespread application of herbal medicine, the WHO has encouraged to use medicinal plants for the treatment of diarrhea [11]. Also, because of its fewer complications, medicinal plants have been implemented as alternative therapy in different countries for thousands of years [12].

*Cuscuta reflexa* Roxb. is such a medicinal herb belonging to the family Convolvulaceae, commonly known as Dodder (English), Swarnalata (Bengali), Akashabela or Amarabela (Hindi) and Akakhilata (Assamese) [13]. It is found in tropical and temperate regions such as India, Malaysia, Thailand, Nepal, and Afghanistan [13,14]. The tribes of Bangladesh and Nepal use this herb to treat pain, edema, and tumor, to maintain the hepatic system, to cure jaundice and skin infections [15,16]. It is also traditionally used for the treatment of insanity, melancholy, diaphoretic, demulcent, fits, and fever [14,17]. Previous research suggests that *C. reflexa* has been found to possess several activities, including antioxidant, antibacterial, antispasmodic, hypoglycemic, hemodynamic, antihypertensive, antiviral, and anticonvulsant [18,19,20,21]. Additionally, a wide range of bioactive constituents has been isolated from this herb, including kaempferol, astragalin, myricetin, quercetin, isorhamnetol, linoleic acid, oleic acid, stearic acid, palmitic acid, *ß*-sitosterol, luteolin, coumarin, *n*-hentriacontane, *α*-amyrin, and sesamin [18]. Although *C. reflexa* holds extensive traditional uses, some of the pharmacological activities of this herb are yet to be investigated. Therefore, the present study is aimed to explore in vivo neuro-pharmacological, anti-nociceptive, and antidiarrheal activities of methanolic extract of *Cuscuta reflexa*. Furthermore, the isolated bioactive compounds from this herb were evaluated for the selected activities through *in* silico approaches, namely molecular docking and pharmacokinetic properties, including absorption, distribution, metabolism, excretion, and toxicity (ADME/T).

## 2. Materials and Methods

### 2.1. Identification and Preparation of Plant Extract

The *Cuscuta reflexa* Roxb. leaves were collected from Noakhali, and the plant was authenticated by Dr. Shaikh Bokhtear Uddin, Taxonomist and Professor, Department of Botany, University of Chittagong with a reference number (SUB 3219) which has been deposited at the Department of Pharmacy, International Islamic University, Chittagong, Bangladesh and also in the Herbarium of the University of Chittagong for future reference. To prepare the crude extract, the grinded powder (250g) was soaked in sufficient methanol (700 mL) for 7 days and vigorously shaken. Then, the entire mixture solutions were filtered and evaporated by using the rotary evaporator to obtain the sticky semi-solid mass which was reserved in 4 °C.

### 2.2. Chemicals

Methanol, acetic acid, and formalin were obtained from Merck (Darmstadt, Germany). Diazepam, imipramine hydrochloride, Loperamide and diclofenac sodium were obtained from Square Pharmaceuticals Ltd. All other chemicals used in this research were of analytical reagent grade until unless specified with additional reference.

### 2.3. Experimental Animals and Ethical Statements

Adult Swiss Albino mice weighing 25–30 g of both male and female were obtained from Jahangir Nagar University, Savar, Bangladesh. All experimental protocols (Pharm-P&D-61/08′19-127) were approved by the institutional animal ethics committee of Department of Pharmacy, International Islamic University Chittagong, Bangladesh.

### 2.4. Acute Oral Toxicity Test

The acute oral toxicity test was performed using standard laboratory conditions according to the “Organization for Environmental Control Development” guidelines (OECD: Guidelines 420; Fixed Dose Method).

### 2.5. Experimental Design (Drugs and Treatments)

Experimental animals (24 mice for each experiments) were separated into four groups (control, standard, and test groups) containing six mice in each section. The test groups were administrated MECR at doses of 200 and 400 mg/kg, b.w, p.o, respectively, whereas the control group received vehicle (1% Tween 80 in water, 10 mL/kg, p.o). The standard drug diazepam (1 mg/kg, b.w, i.p) was used in elevated plus maze test and hole-board test, while imipramine hydrochloride (10 mg/kg, b.w, i.p) was used for tail suspension test (TST) and forced swim test (FST). The diclofenac sodium (10 mg/kg b.w, p.o) was administrated to the mice for an acetic acid induced writhing and formalin induced hind paw licking test. In addition, loperamide (5 mg/kg) was used for the castor-oil induced anti-diarrheal test. Importantly, the reference drugs (diazepam, imipramine hydrochloride, diclofenac sodium, and loperamide) were administrated at 15 min and MECR (200 and 400 mg/kg) or vehicle at 30 min prior to the experiments.

### 2.6. Anxiolytic Activity

#### 2.6.1. Elevated Plus Maze Test (EPM)

The elevated plus maze (EPM) test was performed to investigate the anxiolytic activity of MECR in mice [22]. The apparatus (situated above 40 cm of the floor) used in this test contained two open arms (5 × 10 cm) and two closed arms (5 × 10 × 15 cm) which together merged in a center platform (5 × 5 cm) and exposed as symbol of plus sign. The randomly distributed animals (n = 6) of each group were administrated as mentioned in Section 2.5. After thirty minutes, each treated animal was kept in the midpoint of platform, facing to the closed arms and allowed for free roaming for 5 min. During exploration, open arms entrance and total time spent were recorded.

(1) $$\begin{array}{c} \%\;\mathrm{of}\;\mathrm{entries}\;\mathrm{in}\;\mathrm{the}\;\mathrm{open}\;\mathrm{arm}\\ \;\;\;\;\;\;\;\;\;\;\;\;\;\;\;\;\;\;\;\;\;\;\;\;=\frac{\mathrm{No}.\;\mathrm{of}\;\mathrm{entries}\;\mathrm{in}\;\mathrm{the}\;\mathrm{open}\;\mathrm{arm}}{\mathrm{No}.\;\mathrm{of}\;\mathrm{entries}\;\mathrm{in}\;\mathrm{the}\;\mathrm{open}\;\mathrm{arm}+\mathrm{No}.\;\mathrm{of}\;\mathrm{entries}\;\mathrm{in}\;\mathrm{the}\;\mathrm{closed}\;\mathrm{arm}}\\ \;\;\;\;\;\;\;\;\;\;\;\;\;\;\;\;\;\;\;\;\;\;\;\;\times 100 \end{array}$$

#### 2.6.2. Hole-Board Test for Exploratory Behaviour in Mice (HBT)

In this test, a grid-pattern likes sixteen holes (diameter 3 cm) contained flat platform with enclosed space (40 × 40 × 25 cm) was used as an experimental apparatus which was set up 25 cm above the floor [23]. Dosing treatments for each group of animal are described in Section 2.5. Thirty minutes after post administration of test dose, the experimental animal was situated on the middle of the board and allowed to free movement. Finally, head dipping number through the holes by mice were counted for 5 min.

### 2.7. Antidepressant Activity

#### 2.7.1. Forced Swim Test (FST)

The force swimming test was carried out to evaluate the antidepressant activity of MECR in mice as previously described method [24]. This experiment was performed in two sessions, for instance, preliminary test was conducted the day before the final experiment in order to adapt the animals with environment. A transparent glass tank (25 × 15 × 25 cm) filled up to 15 cm with water (25 ± 1 °C) was used as an experimental apparatus for swimming. Mice of all groups were treated (tests dose, standard drug and vehicle) as mentioned in Section 2.5. After thirty munities, each mouse was placed in the tank for 6 min where first 2 min was considered as initial adjustment time and the next 4 min was recorded as the immobility duration.

#### 2.7.2. Tail Suspension Test (TST)

The tail suspension test is simple and most reliable method to screen the antidepressant activity of MECR [25]. After the administration of all doses, as described in Section 2.5, mice were induced into a state of depression (immobility), hanging by the end of tail using adhesive tape (nearly 1 cm from the tip of the tail). The total time of immobility was recorded during the last four minute of a total six minute for each mouse of all groups.

### 2.8. Anti-Nociceptive Activity

#### 2.8.1. Acetic Acid-Induced Writhing Test

This test has been conducted to evaluate the anti-nociceptive activity of MECR in mice, followed by the method of Adnan et al. [26]. The randomly divided experimental mice (n = 6) of each group were treated as described in Section 2.5 above. After 30 min of treatment, acetic acid 0.6% (v/v) was intraperitoneally injected in mice to induce writhing (abdominal pain). Five min after administration of acetic acid, the writhing number for each mouse was counted for 20 min. The percentage of inhibition was measured by using following equation:

(2) $$\mathrm{Inhibition}\;\left(\%\right)\mathrm{of}\;\mathrm{writhing}=\frac{\mathrm{Total}\;\mathrm{number}\;\mathrm{of}\;\mathrm{writhing}\;\left(\mathrm{control}-\mathrm{test}\;\mathrm{group}\right)}{\mathrm{Total}\;\mathrm{writhing}\;\mathrm{number}\;\mathrm{of}\;\mathrm{the}\;\mathrm{control}}\times 100$$

#### 2.8.2. Formalin Induced Licking Test

In this test, the allocated experimental mice of various groups were administrated as described in Section 2.5. Thirty minutes later of all treatment, a formalin solution (2.5%, v/v, 20 µL) was injected in sub-plantar region of hind paw of mice to induce pain. Mice reflected pain response by licking and biting the hind paw (the injected area) which was counted during both the first phases (5 min) and the late phase (15–30 min). The percentage of inhibition of licking time was calculated as described in [26].

### 2.9. Anti-Diarrheal Activity

#### Castor Oil-Induced Diarrhea

The antidiarrheal activity of MECR was performed according to the method described previously [27]. The allocated mice were fasted for 24 h and dosing treatments for each group of mice (n = 6) were described in Section 2.5. Mice were orally treated with castor oil (0.5 mL) and individually placed in the blotting paper cage after 1 h of administration. Each 1 h after, blotting paper was changed and the numbers of both dry and wet faeces excreted by the mice were counted during the 4-h observation period. The percent of inhibition of defecation and diarrhea are counted by using following equation:

(3) $$\begin{array}{c} \mathrm{Inhibition}\;\left(\%\right)\mathrm{of}\;\mathrm{Diarrhea}\\ \;=\frac{\mathrm{Total}\;\mathrm{number}\;\mathrm{of}\;\mathrm{diarrheal}\;\mathrm{faces}\;\left(\mathrm{control}-\mathrm{test}\;\mathrm{group}\right)}{\mathrm{Total}\;\mathrm{number}\;\mathrm{of}\;\mathrm{diarrheal}\;\mathrm{faces}\;\mathrm{of}\;\mathrm{the}\;\mathrm{control}}\times 100 \end{array}$$

## 3. In Silico Studies

### 3.1. Molecular Docking Analysis: Ligand Preparation

The chemical structures of fifteen major compounds of MECR were downloaded from PubChem compound repository (https://pubchem.ncbi.nlm.nih.gov/). The ligand was prepared by using the LigPrep tool, which embedded in Schrödinger suite-Maestro v 10.1, where the following parameters were used as follows: neutralized at pH 7.0 ± 2.0 using Epik 2.2 and the OPLS_2005 force field were used for minimization.

#### 3.1.1. Molecular Docking Analysis: Enzyme/Receptor Preparation

Three-dimensional crystallographic structures of enzyme/receptors were obtained from the Protein Data Bank RCSB PDB [28] potassium channel receptor (PDB: 4UUJ) [29], human serotonin receptor (PDB: 5I6X) [30], cyclooxygenase-1 (COX-1, PDB: 2OYE) [31], cyclooxygenase-2 (COX-2, PDB: 3HS5) [32], M3 muscarinic acetylcholine receptor (PDB: 4U14) [33], and 5-HT3 receptor (PDB: 5AIN) [34]. The enzyme/receptor was prepared for a docking experiment using Protein Preparation Wizard, which embedded in Schrödinger suite-Maestro v 10.1.

#### 3.1.2. Molecular Docking Analysis: Glide Standard Precision Docking

A molecular docking study was performed to reveal the possible mechanism of action of the selected compounds behind the biological activities of the MECR against the respective enzymes/receptor for an anxiolytic, antidepressant, anti-nociceptive, and antidiarrheal activity. Docking experiments were performed using Glide standard precision docking, which was embedded in Schrödinger suite-Maestro v 10.1, as we described previously [27].

### 3.2. In Silico Study: Determination of Pharmacokinetic Parameters by SwissADME

The pharmacokinetic parameters or drug-likeness properties of the selected compounds were determined by SwissADME online tool (http://www.swissadme.ch/). In the present study, an orally active drug should fulfill the following drug-likeness parameters to demonstrate their pharmaceutical fidelity such as molecular weight of the compounds, Lipophilicity (LogP), the number of hydrogen-bond acceptors, and the number of hydrogen-bond donors based on the Lipinski’s rule.

### 3.3. In Silico Study: Toxicological Properties Prediction by admetSAR

Toxicological properties of the selected compounds were determined using the admetSAR online tool (http://lmmd.ecust.edu.cn/admetsar1/predict/) sine toxicity is a prime concern during the development of new drugs. In the present study, Ames toxicity, carcinogenic properties, acute oral toxicity, and rat acute toxicity were predicted.

## 4. Statistical Analysis

The data were expressed as mean ± standard error of mean (SEM). Statistical comparisons were performed using one-way ANOVA followed by Dunnett’s multiple comparison tests. The values obtained were compared with the vehicle control group and were considered statistically significant when *p* < 0.05.

## 5. Result

### 5.1. Elevated Plus Maze (EPM)

**Figure 1:**
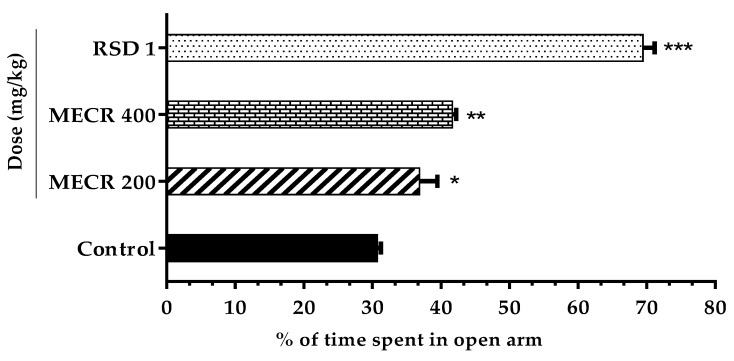
The effect of methanol extract of *Cuscuta reflexa* (MECR) in EPM test in mice. Values are expressed in mean ± SEM (n = 6). * *p* < 0.05, ** *p* < 0.01 and *** *p* < 0.001 compared with the control group (Dunnett’s test). RSD: reference standard drug (Diazepam, 1 mg/kg).

Figure 1 illustrated the potential effect of MECR in EPM test. Administration of MECR (400 and 200 mg/kg) demonstrated a dose depend increase of locomotion (spending time in the open arms). Particularly, 400 mg/kg significantly (*p* < 0.01) elevated the percentage of time spent in the open arms (41.66 ± 0.56), whereas 200 mg/kg responded a moderate (36.86 ± 2.62) but significant (*p* < 0.05) anxiolytic influence compared to the control group. In contrast, diazepam (reference standard drug, at 1 mg/kg, i.p.) treated mice exposed a marked acceleration (*p* < 0.001) in the percentage of open arms time spent (69.46 ± 1.70).

#### Hole Board Test (HBT)

**Figure 2:**
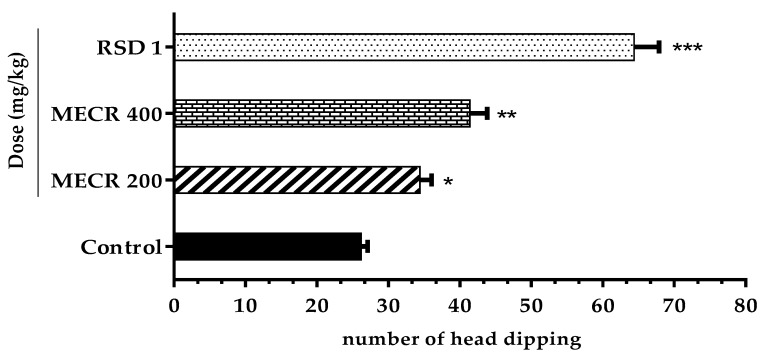
The effect of methanol extract of *Cuscuta reflexa* (MECR) in hole board test in mice. Values are expressed in mean ± SEM (n = 6). * *p* < 0.05, ** *p* < 0.01 and *** *p* < 0.001 compared to control (Dunnett’s test). RSD: reference standard drug (Diazepam, 1 mg/kg).

In this test, mice treated with MECR (200 and 400 mg/kg) exhibited notable exploratory behavior in a dose-dependent fashion (Figure 2). Particularly, the treatment of 400 mg/kg revealed a significant (*p* < 0.001) hole poking tendency followed by a higher number of head dipping (41.40 ± 2.42) compared to 200 mg/kg (34.40 ± 1.63); (*p* < 0.05). In addition, the positive control diazepam (1 mg/kg, i.p.) revealed (*p* < 0.001) higher number of head dipping (64.33 ± 2.32) in comparison to the control group (26.33 ± 1.44).

### 5.2. Forced Swimming Test (FST)

**Figure 3:**
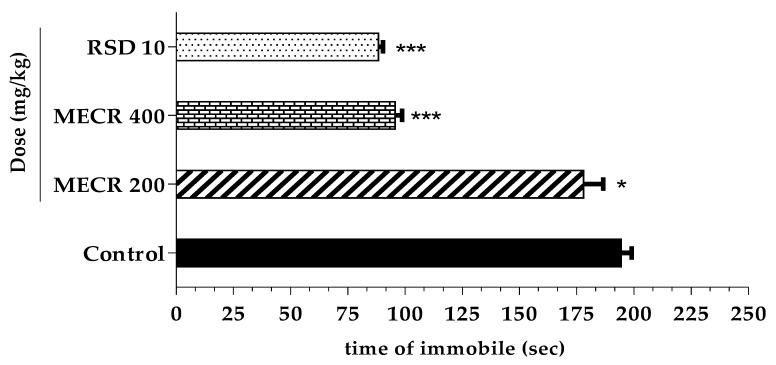
The effects of methanol extract of *Cuscuta reflexa* (MECR) in forced swimming test in mice. Values are expressed in mean ± SEM (n = 6). * *p* < 0.05 and *** *p* < 0.001 compared to control (Dunnett’s test). RSD: reference standard drug (Imipramine hydrochloride, 10 mg/kg).

The antidepressant efficiency of MECR (200 and 400 mg/kg) studied by force swimming test was displayed in Figure 3. Over the observation period during FST test, the depressive behavior, such as time of immobility (sec) was decreased in a significant dose-dependent manner. Mice administrated with 400 mg/kg apparently manifested worth mentioning (*p* < 0.001) immobile time (95.6 ± 3.14 s) which was analogous (*p* < 0.001) to the immobile time (88.3 ± 2.07 s) of imipramine (10 mg/kg), used as a reference (standard) antidepressant. In contrast, 200 mg/kg also showed a slight decrease in time of immobility (178 ± 8.60 s) (*p* < 0.05) as compared to control group (194.4 ± 4.57 s). In addition, the percentage of decrease in immobility (depression) for imipramine, MECR 200, and 400mg/kg was estimated 54.57, 8.43 s, and 50.82% respectively.

####  Tail Suspension Test (TST)

**Figure 4:**
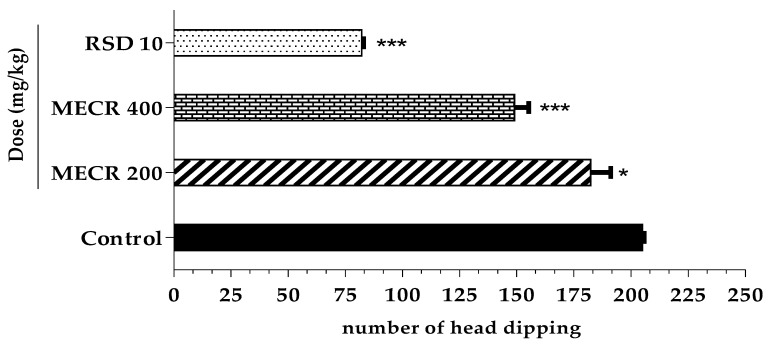
The effect of methanol extract of *Cuscuta reflexa* (MECR) in tail suspension test in mice. Values are expressed in mean ± SEM (n = 6). * *p* < 0.05 and *** *p* < 0.001 compared to control (Dunnett’s test). RSD: reference standard drug (Imipramine hydrochloride, 10 mg/kg).

Outcome of tail suspension test is shown in Figure 4. In this test, treatment of MECR at both (200 and 400 mg/kg) doses exhibited antidepressant like action. Both doses demonstrated significant reduction of immobility time by 11.02% (*p* < 0.05) and 27.31% (*p* < 0.001), respectively. The decreased immobile time was recorded 182.4 ± 8.70 s for 200 mg/kg and 149 ± 6.25 s for 400 mg/kg in comparison to control 205 ± 0.70 s. However, standard drug imipramine (10mg/kg) revealed a significant (*p* < 0.001) decrease of immobile time (82.2 ± 0.86 s) with a 59.90% antidepressant effect.

### 5.3. Acetic Acid-Induced Writhing Test

**Figure 5:**
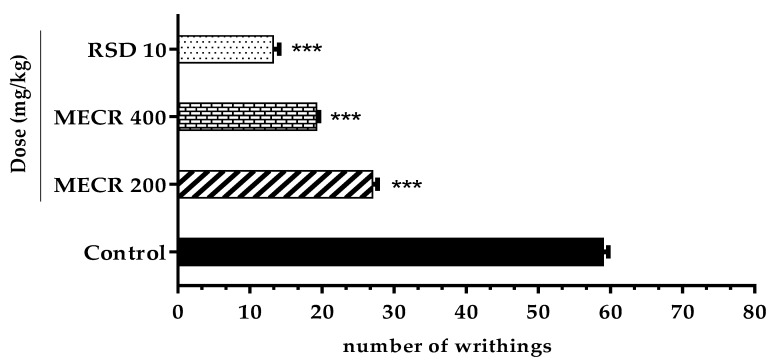
The effect of methanol extract of *Cuscuta reflexa* (MECR) in acetic acid-induced writhing test in mice. Values are expressed in mean ± SEM (n = 6). *** *p* < 0.001 compared to control (Dunnett’s test). RSD: reference standard drug (Diclofenac sodium, 10 mg/kg).

Figure 5 represents the inhibitory potential of MECR against acetic acid induced abdominal (writhing) pain in mice. The writhing extent was remarkably suppressed by the outstanding anti-nociceptive response of MECR at both doses (400 and 200 mg/kg). Writhing numbers and percentage of writhing inhibition for the doses of 200 and 400 mg/kg were counted 27 ± 0.70 (54.23%) and 19.2 ± 0.37 (67.45%) respectively, compared to the control (59 ± 0.70) group. The most effective writhing inhibition was noted 77.62% with mean writhing number 13.2 ± 0.86 (*p* < 0.001) for the reference standard drug “diclofenac sodium” (10 mg/kg).

#### Formalin Induced Licking Test

Both doses (200 and 400 mg/kg) of MECR significantly (*p* < 0.001) attenuated formalin induced neurogenic (5 min) and inflammatory (15–30 min) phases of the pain response in mice (Table 1). During the early (neurogenic pain) phase, paw licking duration for 400 mg/kg was recorded as 31.00 ± 0.70 s, with pain inhibition of 45.61%, while late (inflammatory pain) exposed 50.00% pain inhibition with licking duration of 20.80 ± 1.77 s. Similarly, dose of 200 mg/kg alleviated both neurogenic and inflammatory pain by 33.33% and 37.50%, respectively. The standard drug (diclofenac sodium 10 mg/kg) also produced obvious pain inhibition by 73.85% in the early phase and 67.30% in the late phase.

### 5.4. Castor Oil-Induced Diarrhea

The outcomes following various doses of MECR (200 and 400 mg/kg) on castor oil-induced diarrhea are summarized in Table 2. A dose dependent inhibition of diarrhea was observed, while 400 mg/kg demonstrated maximum inhibition (55.12%, *p* < 0.001), which was higher than the anti-diarrheal activity of standard drug loperamide 53.84% (*p* < 0.001). The number of defecation was also significantly (*p* < 0.001) reduced for 400 kg/mg (7.00 ± 0.44), with an inhibition of 51.38% defecation compared to the control, whereas loperamide showed 73.61% inhibition of defecation.

### 5.5. Molecular Docking Study for Anxiolytic and Antidepressant Activity

In the case of the anxiolytic docking study, fifteen selected compounds from *Cuscuta reflexa* were docked against the potassium channel (PDB: 4UUJ) receptor and displayed the docking scores ranging from +0.71 to −6.42 kcal/mol. The results of the docking study are shown in Table 3, and the docking figures are presented in Supplementary Figures S1 and S2. From the results, it was observed that the compounds Myricetin (−6.42 kcal/mol) exposed the highest score against target receptor, followed by quercetin (−5.75 kcal/mol), isorhamnetol (−5.56 kcal/mol), kaempferol (−5.55 kcal/mol), luteolin (−5.35 kcal/mol), astragalin (−4.94 kcal/mol), coumarin (−4.56 kcal/mol), sesamin (−3.88 kcal/mol), *ß*-sitosterol (−3.63 kcal/mol), *α*-amyrin (−2.70 kcal/mol), *n*-hentriacontane (−2.18 kcal/mol), and linoleic acid (+0.71 kcal/mol). The reference standard drug, diazepam, showed the docking score −3.73 kcal/mol against potassium channel (PDB: 4UUJ) receptor. Conversely, for antidepressant docking analysis, our study demonstrated that astragalin and palmitic acid have the highest and lowest binding affinity against the human serotonin receptor (PDB: 5I6X) with a docking score of −9.23 kcal/mol and +0.94 kcal/mol respectively. The ranking order of docking score for antidepressant effect is as follow: astragalin > myricetin > isorhamnetol > sesamin > quercetin > kaempferol > luteolin > coumarin > linoleic acid > oleic acid > stearic acid > palmitic acid. The reference standard drug, imipramine hydrochloride exposed the docking score −8.11 kcal/mol against the human serotonin receptor (PDB: 5I6X).

#### 5.5.1. Molecular Docking Study for Anti-Nociceptive Activity

The results of anti-nociceptive docking study are presented in Table 3, and the docking figures are displayed in Supplementary Figures S3 and S4. The present study exhibited that kaempferol and palmitic acid have the upmost and lowest binding affinity against the COX-1 enzyme (PDB: 2OYE) with a docking score of −8.61 kcal/mol and −1.66 kcal/mol respectively. The ranking order of docking score is given below: kaempferol > quercetin > sesamin > luteolin > coumarin > isorhamnetol > myricetin > astragalin > oleic acid > linoleic acid > stearic acid > palmitic acid. On the other hand, luteolin and palmitic acid demonstrated the highest and lowest binding affinity against the COX-2 enzyme (PDB: 6COX) with a docking score of −8.58 kcal/mol and +0.18 kcal/mol respectively. The ranking order of docking score is as follow: luteolin > isorhamnetol > quercetin > coumarin > kaempferol > sesamin > myricetin > stearic acid > oleic acid > linoleic acid > palmitic acid. The reference standard drug, diclofenac-Na, manifested the binding affinity against COX-1 and COX-2 enzymes are −7.31 kcal/mol and −7.71 kcal/mol, respectively.

#### 5.5.2. Molecular Docking Study for Antidiarrheal Activity

**Figure 6:**
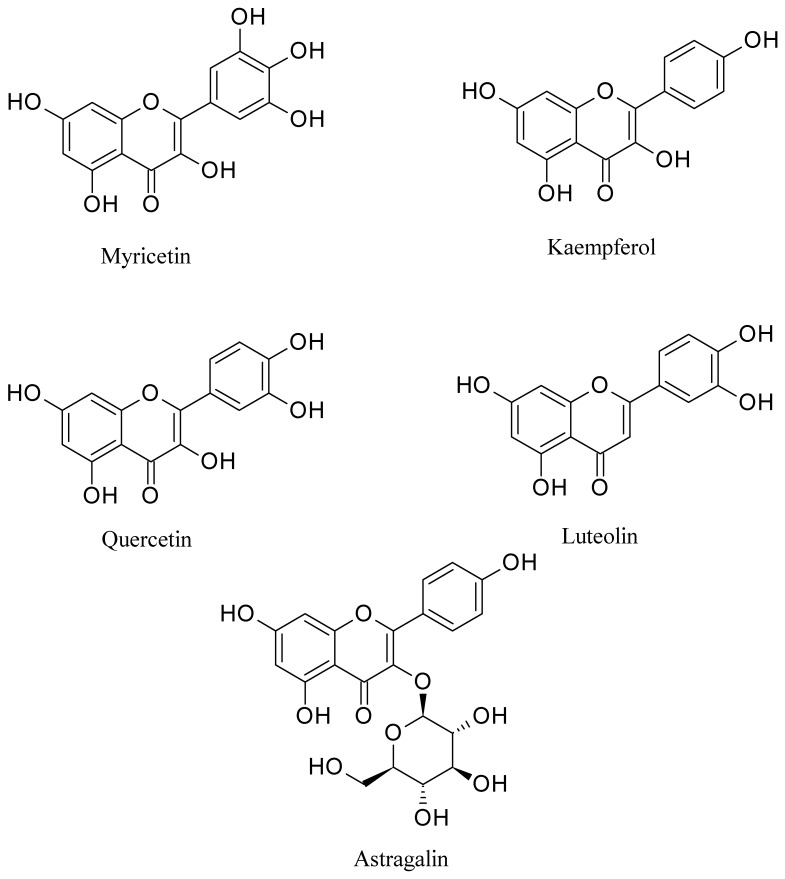
Chemical structures of major bioactive compounds identified in the MECR.

In this case, astragalin and palmitic acid have demonstrated the maximum and lowermost binding affinity against M3 muscarinic acetylcholine receptor (PDB ID: 4U14) with a docking score of −9.88 kcal/mol and −1.88 kcal/mol respectively. The ranking order of docking score is presented below: astragalin > luteolin > kaempferol > isorhamnetol > quercetin > myricetin > coumarin > sesamin > linoleic acid > oleic acid > stearic acid > palmitic acid. Loperamide (reference drug) exposed −7.39 kcal/mol binding affinity against M3 muscarinic acetylcholine receptor (PDB ID: 4U14). In contrast, quercetin and stearic acid displayed the highest and lowest binding affinity against 5-HT3 receptor (PDB ID: 5AIN) with the docking score of −6.13 kcal/mol and −0.63 kcal/mol, respectively. The ranking order of docking score is presented: quercetin > coumarin > kaempferol > isorhamnetol > myricetin > luteolin > sesamin > linoleic acid > oleic acid > palmitic acid > stearic acid. The results of the docking study are shown in Table 3, and the docking figures are presented in Supplementary Figures S5 and S6. However, based on the highest docking score against all investigated receptors, five major compounds were documented (Figure 6) and their binding interactions are presented in Table 4.

### 5.6. Pharmacokinetic (ADME) and Toxicological Properties Prediction

Per Lipinski’s rules of five, the pharmacokinetic properties of the selected compounds were calculated using an online tool, SwissADME. Here, Lipinski stated that a drug/compound would be orally bioavailable if it follows the following criteria such as molecular weight < 500 amu, Hydrogen bond acceptor sites < 10, Hydrogen bond donor sites < 5, and Lipophilicity value LogP ≤ 5. Result of the present study exhibited that all the compounds, except “astragalin”, satisfied Lipinski’s rules, which indicates that these compounds have good oral bioavailability (Table 5).

In addition, toxicological properties of the five selected compounds were also predicted using admetSAR online server, where our study showed that the selected compounds were non-Ames toxic, non-carcinogenic, and demonstrated weak rat acute toxicity properties (Table 6).

## 6. Discussion

Neurological disarrays such as anxiety and depression are the most common emotional disorders and represent an ever-cumulative intimidation to public health [35]. It is suggested by different corroborations that depressive and anxiety disorders correspond to and do not generate individually distinct disease entities [36]. Although the particular etiology of anxiety and depression remains in a great mystery, one of the most possible stimulators of these syndromes is chronic pain, which has an extreme reciprocal correlation with anxiety and depression [37]. Interestingly, the clinical expressions, neurotransmitters, pro-inflammatory cytokines, and neurological pathways of pain and depression have parallel manifestation [38]. Moreover, the mechanisms of neurotransmitters such as serotonin and norepinephrine, have an analogous role for modulating depression and pain signaling pathways in the brain and nervous system [39]. Hence, patients with such chronic pain may suffer from anxiety, accompanied by a progressive depressive state.

Currently, selective serotonin and/or serotonin-norepinephrine reuptake inhibitors along with voltage-dependent calcium channels *α*2*δ* subunit ligands are considered as top-line synthetic antidepressant drugs of choice for treating chronic pain [40]. Likewise, standard pharmaceutical agents, some potential herbs such as California poppy, Saint John’s Wort, Passionflower, Kava, and Saffron have been suggested for the management of anxiety, depression, and neurological based chronic pain [41]. However, to develop classical lead compounds from a medicinal plant having manifold pharmacological actions, the implementation of innovative animal prototypes and well-validated tests are inevitable to get a reliable preclinical and clinical conclusion [42]. Hence, we have designed in vivo preclinical investigations whether *Cuscuta reflexa* leaves or its isolated components have the multifaceted pharmacological possessions in mitigating the neuropsychiatric disorders and neurobiological based protracted pain. Therefore, we have assessed anxiolytic, antidepressant, anti-nociceptive, and antidiarrheal potentials of methanol extract of *Cuscuta reflexa* leaves, utilizing suitable animal research models which accurately reflect various aspects of human psychopathology.

To verify the anxiolytic activity of MECR, we utilized elevated plus maze (EPM) and hole board (HB) animal models [43,44]. Both tests are regarded as popular paradigms due to the quick valuation of the possible anxiety modulating responses in mice. The typical EPM tool has two opposite open and two bounded arms, whereas the open arena is supposed to be more fearful for the animals, and an anxiolytic agent stimulates the mice to open arm exploration [45]. Similarly, the hole board test (HBT) has been designed to measure the exploratory responses and multiple dimensions of the unconditioned behavior of a mouse to an unfamiliar environment [46]. The manifestation of more hole poking (head dipping) tendency indicates a high level of anxiolytic activity, while the hesitancy expression of visiting the hole results from a high level of anxiety. However, during the experiment, mice treated with MECR (200 and 400 mg/kg) demonstrated a reduction in anxiety-like behavior by reflecting increased entries to, and time spent in the open alleys as well as elevated the exploratory behavior followed by demonstrating the higher number of head dipping. The neurobiological mechanism of anxiety is the result of either an imbalance of neurotransmitter (dopamine, GABA, and serotonin) function or dysregulation of glutamatergic, serotonergic, GABA-ergic, and noradrenergic transmission [47]. In our experiment, MECR may exert anxiolytic actions by modifying neurotransmitter synthesis and functions. It is supposed that active components of MECR interact with the neurotransmitter or neuromodulator receptors, which regulate the neuronal communication, stimulate the CNS activity, and improve the function of endocrine systems [48].

Some potential anxiolytic herbs, such as *R. rosea* and *C. sativus* have been proven to have antidepressant activity [49]. This bilateral neuropharmacological action has been known as “halo effect,” of which once anxiety is cured efficiently, depression may also be de-escalated [50]. Generally, the dysfunction of the corticotrophin-releasing factor (CRF) due to over activation of the hypothalamic-pituitary-adrenal (HPA) axis causes the manifestation of depressive symptoms [51]. Nevertheless, effective antidepressant treatment suppresses the stress-induced HPA axis activation, followed by restoring the rational expression and function of CRF [52]. In our study, MECR was further subjected to a force swimming test (FST) and tail suspension test (TST) to explore antidepressant-like activity as well as the pathological mechanism of depression. The ambient of FST and TST are very stressful, where without using antidepressant agent, mice reflect a state of behavioral despair e.g., immobility (passive behavior). However, MECR (200 and 400 mg/kg) treatment in mice revealed dynamic behaviors, such as decreased immobility in swimming test and increased struggling tendency in tail suspension test. These noteworthy antidepressant-like effects of MECR might be either due to the inhibition of monoamine reuptake or significant suppression of HPA axis activation.

Numerous studies on animal models have reported that antidepressant treatments (tricyclic antidepressants and serotonin noradrenaline reuptake inhibitors) are beneficial to reduce protracted pain, such as neuropathic pain [38]. It was found that the accumulation of noradrenaline in the spinal cord by reuptake inhibition can efficiently suppress neuropathic pain through α2 adrenergic receptors. Although the precise mechanisms of antidepressants underlying treatment of persistent pain remain uncertain, currently available preclinical and clinical outcomes revealed that several neurotransmitters, neuromodulators, mainly, serotonin-norepinephrine reuptake inhibitors may be the most viable mediators for the modulation of prolonged pain like neuropathic pain symptoms [53]. Meanwhile, the anxiolytic and antidepressant activity of MECR has been found in a promising level; and importantly, *C. reflexa* is used for the treatment of low back pain in traditional medicine. Therefore, we intended to explore the anti-nociceptive action of MECR further.

To evaluate peripherally reactive analgesics, we investigated acetic acid stimulated writhing test in mice. Induction of acetic acid causes immediate liberation of endogenous pain mediators; also, various visceral pro-inflammatory mediators or chemo-sensitive nociceptors are activated and released, which further enter into the dorsal horn of the central nervous system by aggravating peripheral neurons [26]. This mechanism of acetic acid is responsible for abdominal constriction (visceral pain) and pain sensation followed by the manifestation of writhing in mice [54]. However, during our experiment, MECR (200 and 400 mg/kg) dose-dependently inhibited abdominal constriction in mice, resulting in notable anti-nociceptive activity, which perhaps due to the presence of potential analgesic components in MECR inhibiting the release of pain mediators.

Although the anti-nociceptive effects of MECR in the writhing test does not delineate whether central or peripheral processes mediate the responses. Hence, we conducted a formalin induce leaking test in order to clarify neurogenic and inflammatory pain responses [26]. Formalin (intra-planter injection) triggers incessant nociception characterized by a biphasic pain reaction [55]. The initial phase (0–5 min) is associated with the acute nociceptive neurogenic pain, possibly due to provocation of sensory C and A*δ* fibers through neuropeptide, for instance, substance P. The late phase (15–30 min) relates to inflammatory nociception which stimulates to release various chemical mediators, such as histamine, serotonin, amino acids, prostaglandins, and bradykinin. Importantly, drugs that have the central effect can inhibit the mediators of both phases, while peripherally acting drugs suppress only the mediators of the late phase [54,56]. In this regard, MECR suppressed both phases of mediators, confirmed by the substantial anti-nociceptive activity in a dose-dependent fashion, which indicates that MECR possesses the potential phytoconstituents with central effects.

During the antidiarrheal test, the administrated castor oil (0.5 mL, natural laxative) releases its active metabolite “ricinoleic acid” through the action of small intestinal lipase [57]. The liberated ricinoleic acid then binds with EP3 prostanoid receptors on smooth muscle cells, which suppress electrolytes and water absorption from the intestine, causing dysfunction and noxious effect in the intestine [58]. In addition, ricinoleic acid induces local inflammation in the intestine through stimulation of various pro-inflammatory mediators, such as prostaglandin biosynthesis, which also responsible for the inhibition of water and ions reabsorption [59]. Any antidiarrheal agent like loperamide accelerates the absorption rate, followed by reducing movement and volume of intestinal contents [27]. Analagous to this mechanism, MECR at all doses showed a dose-dependent antidiarrheal action, demonstrated by a significant inhibitory response, in terms of both defecation rate and diarrhea.

During comprehensive analyses, MECR at 400 mg/kg was found to be most effective in all experiments which might be due to the combined action of important phytoconstituents, in addition to those isolated and potentially other as yet uncharacterized bioactive compounds possess in MECR. However, for the practical implementation of this dose to the human purpose, it is necessary to identify and isolate the pure components responsible for the observed biological effects. In addition, in acute toxicity test, all measured doses (10 to 1500 mg/kg) did not expose any noticeable indication of toxicity, behavioral abnormalities, and potential defects on motor activities (excitability and sedation). Moreover, overt toxicological effects, particularly physical changes (allergic reaction and loss of body weight), were not observed, which confirmed that MECR has no toxic effects up to 1500 mg/kg.

Molecular docking is a vital tool in structural molecular biology and computer-assisted drug design (CADD). This tool contributes to predicting the binding mode of active compounds against the essential proteins [60]. Additionally, it is used to comprehend the possible molecular mechanism of actions of various pharmacological activities [27]. However, to correlate with the findings of our current experimental results, molecular docking was performed to understand the molecular mechanism better. In this study, fifteen major compounds of *Cuscuta reflexa* were examined against six target receptors or enzymes, namely, potassium channel receptor (PDB: 4UUJ), human serotonin receptor (PDB: 5I6X), cyclooxygenase-1 (COX-1, PDB: 2OYE) and cyclooxygenase-2 enzymes (COX-2, PDB: 3HS5), M3 muscarinic acetylcholine receptor (PDB ID: 4U14), and 5-HT3 receptor (PDB ID: 5AIN). Among the fifteen, eleven phytocompounds (kaempferol, astragalin, myricetin, quercetin, isorhamnetol, linoleic acid, *ß*-sitosterol, luteolin, coumarin, *α*-amyrin, and sesamin) were found which docked against both the potassium channel receptor (PDB: 4UUJ) and human serotonin receptor (PDB: 5I6X) for anxiolytic and antidepressant activity respectively. The anxiolytic and antidepressant activity of the MECR might thus be explained by the presence of kaempferol [61], quercetin [62], isorhamnetol [63], linoleic acid [64], coumarin [65], *α*-amyrin [66], and sesamin [67], which manifested excellent docking scores and for which bioactivities have previously been reported.

The same eleven phyto-compounds were also detected in common, which docked against both COX-1 and COX-2 enzymes. It has been previously reported that kaempferol [61], myricetin, quercetin [62], isorhamnetol [63], linoleic acid [64], oleic acid [68], stearic acid [69], palmitic acid [70], luteolin [71], coumarin [65], sesamin [67] possess analgesic and anti-inflammatory properties, which perhaps the possible reason for the anti-nociceptive activity of MECR.

In the antidiarrheal docking analysis, similar (eleven) phyto-compounds also docked against both M3 muscarinic acetylcholine receptor and 5-HT3 receptor, such as kaempferol, myricetin, quercetin, isorhamnetol, linoleic acid, oleic acid, stearic acid, palmitic acid, luteolin, coumarin, sesamin. From these results, we can conclude that the studied phyto-compounds might, in part, be responsible for the antidiarrheal activity of MECR through interaction with these target receptors.

Furthermore, according to the highest score in the molecular docking study, five compounds have been selected, namely kaempferol, luteolin, myricetin, astragalin, and quercetin, to examine their ADME/T and toxicological properties further. According to Lipinski’s rule of five, orally administered drugs should have a molecular weight < 500 amu, Hydrogen bond acceptor sites < 10, Hydrogen bond donor sites < 5, and Lipophilicity value, LogP ≤ 5. If any drugs/compounds violate all of these rules, it will not be considered as good oral bioavailability [72,73]. Our study exhibited that none of the phyto-compounds except “astragalin” violated these rules, which indicate good oral bioavailability of the documented bioactive compounds. The study of toxicology demonstrated that none of the compounds posed a risk of Ames toxicity, carcinogenicity, acute oral toxicity, and weak rat acute toxicity. Therefore, all five phytocompounds could be considered for promising drug candidates with good oral bioavailability.

## 7. Conclusion

Collectively, the experimental pharmacological evidences support the folkloric value and potentiality of this plant. In this study, MECR has been proved to have noteworthy anxiolytic and antidepressant efficacy. The study also demonstrated that MECR possesses remarkable anti-nociceptive activity which was verified by various pain models. In addition, significant and dose dependent response regarding antidiarrheal activity has enhanced the value of this plant. Furthermore, various identified active compounds from *Cuscuta reflexa* revealed a promising binding attraction towards different proteins in molecular docking analysis. Importantly, the selected active compounds have manifested their drug-like characteristics and safeness in ADME/T and toxicology studies. Overall, *Cuscuta reflexa* can be considered as a sustainable candidate for the development of new drug having multiple pharmacological effects. However, further investigations are required to identify the pure active compounds and their molecular mechanisms also need to be explored for long term safety.

## Figures and Tables

**Table 1:** Anti-nociceptive effect of MECR in formalin induced paw licking test in mice.

Treatment (mg/kg)	Licking Time (s) (Mean ± SEM)			
	Early Phase (0–5 min)	Inhibition (%)	Late Phase (15–30 min)	Inhibition (%)
Control	57.00 ± 1.14	-	41.60 ± 1.02	-
RSD 10	14.90 ± 0.71***	73.85	13.60 ± 0.92***	67.30
MECR 200	38.00 ± 2.02 ***	33.33	26.00 ± 1.09***	37.50
MECR 400	31.00 ± 0.70***	45.61	20.80 ± 1.77***	50.00

Each value is expressed as mean ± SEM (n = 6). *** *P* < 0.001 compared with the control group (Dunnett’s test). MECR, methanol extract of *Cuscuta reflexa* leaves; RSD: reference standard drug (Diclofenac Na, 10 mg/kg).

**Table 2:** The effect of MECR on feces count in castor oil-induced diarrhea in mice.

Treatment (mg/kg)	Total Number of Dry Feces	% of Inhibition of Defecation	Total Number of Diarrheal Feces	% of Inhibition of Diarrhea
Control	14.40 ± 0.74	-	15.60 ± 0.74	-
RSD 5	3.80 ± 1.01***	73.61	7.20 ± 0.37***	53.84
MECR 200	9.60 ± 0.67**	33.33	13.20 ± 1.15	15.38
MECR 400	7.00 ± 0.44***	51.38	7.00 ± 0.83***	55.12

Each value is expressed as mean ± SEM (n = 6). *** *P* < 0.01 and *** *P* < 0.001 compared with the control group (Dunnett’s test). MECR, methanol extract of *Cuscuta reflexa* leaves; RSD: reference standard drug (Loperamide, 5 mg/kg).

**Table 3:** Docking score of the selected isolated compounds of *Cuscuta reflexa*.

Compounds	PubChem ID	Docking Score ^1^					
		2OYE	6COX	4UUJ	5I6X	4U14	5AIN
Kaempferol	5280863	**−8.61**	−6.70	−5.55	−6.94	−7.90	−5.53
Astragalin	5282102	−6.15	-	−4.94	**−9.23**	**−9.88**	-
Myricetin	5281672	−7.03	−4.94	**−6.42**	−7.60	−7.68	−5.18
Quercetin	5280343	−8.52	−7.11	−5.75	−7.13	−7.78	**−6.13**
Isorhamnetol	5281654	−7.75	−8.27	−5.56	−7.56	−7.81	−5.39
Linoleic acid	5280934	−2.12	−0.76	0.71	−2.44	−3.63	−1.19
Oleic acid	445639	−2.84	−1.88	-	−2.39	−3.39	−0.96
Stearic acid	5281	−2.02	−2.69	-	−1.49	−2.00	−0.63
Palmitic acid	985	−1.66	0.18	-	−0.94	−1.88	0.66
*β*-sitosterol	222284	-	-	−3.63	−6.97	-	-
Luteolin	5280445	−8.35	−**8.58**	−5.35	−6.94	−8.44	−5.11
Coumarin	323	−7.91	−7.01	−4.56	−6.84	−7.09	−5.94
*n*-Hentriacontane	12410	-	-	−2.18	-	-	-
*α*-amyrin	73170	-	-	−2.70	−6.34	-	-
Sesamin	72307	−8.52	−6.43	−3.88	−7.15	−6.93	−3.90
**Standard drug:**							
Diclofenac-Na/Diazepam/ Imipramine/Loperamide		−7.31	−7.71	−3.73	−8.11	−7.39	-

^1^ Docking scores in kcal/mol; bold text indicates the highest score.

**Table 4:** Binding interactions of the major five compounds identified in the molecular docking study.

Proteins	Ligands	Hydrogen Bond Interactions		Hydrophobic Interactions	
		Amino Acid Residue	Distance (Å)	Amino Acid Residue	Distance (Å)
2OYE	Kaempferol	SER-530	3.93	ILE-523 VAL-349 ALA-527 LEU-352 GLY-526 TYR-385	5.41 6.15 4.83 5.11 4.45 6.79 2.43
6COX	Luteolin	SER-530 ARG-120 LEU-352 GLN-192	3.14 5.80 3.83 5.82	VAL-349 ALA-527 VAL-523 SER-353	4.61 5.40 5.21 4.03 4.68 4.11 4.03
4UUJ	Myricetin	GLU-62 GLY-53 ARG-57 LEU-86	5.53 4.15 4.88 7.62 4.80	PRO-55 ALA-54	4.97 5.07 4.79
5I6X	Astragalin	THR-497 TYR-175 ASP-98	3.94 3.45 5.77 4.69	TYR-176 TYR-95	7.30 5.18 6.40
4U14	Astragalin	CYS-532 TYR-529 TYR-148 THR-231 ASN-507 ASN-152	4.15 3.10 4.85 4.45 3.82 3.36	TYR-529 ALA-523 CYS-532 TRP-503	7.30 6.80 6.08 4.67 6.03 5.55
5AIN	Quercetin	GLU-191 TRP-145	3.76 4.55	TYR193 TYR186 TRP-145	6.84 7.22 6.68 4.81

**Table 5:** Physicochemical properties of the compounds for good oral bioavailability.

Compounds	Lipinski Rules				Lipinski’s Violations
	MW	HBA	HBD	Log P	
	<500	<10	≤5	≤5	≤ 1
Kaempferol	286.24	6	4	1.58	0
Astragalin	448.38	11	7	-0.25	2
Myricetin	318.24	8	6	0.79	1
Quercetin	302.24	7	5	1.23	0
Luteolin	286.24	6	4	1.73	0

MW, Molecular weight (g/mol); HBA, Hydrogen bond acceptor; HBD, Hydrogen bond donor; Log P, Lipophilicity; NRB: number of rotatable bond; TPSA: topological polar surface area.

**Table 6:** Toxicological properties of the major five compounds identified in the molecular docking study.

Parameters	Compound Name				
	Kaempferol	Luteolin	Myricetin	Astragalin	Quercetin
Ames toxicity	NAT	NAT	NAT	AT	NAT
Carcinogens	NC	NC	NC	NC	NC
Acute oral toxicity	II	II	II	III	II
Rat acute toxicity	3.0825	3.0200	3.0200	2.3869	3.0200

AT: Ames toxic; NAT: Non Ames toxic; NC: Non-carcinogenic; Category-II means (50 mg/kg > LD_50_ < 500 mg/kg); Category-III means (500 mg/kg > LD_50_ < 5000 mg/kg).

## Supplementary Materials

The following are available online at https://www.mdpi.com/1424-8247/13/3/50/s1, **Figure S1.** 2D interactions of the Kaempferol (A), Astragalin (B), Myricetin (C), Quercetin (D), Isorhamnetol (E), Linoleic acid (F), ß-sitosterol, Luteolin (G), Coumarin (H), n-Hentriacontane (I), α-amyrin (J), and Sesamin (K) with the active site of potassium channel (PDB: 4UUJ) receptor for anxiolytic activity. Colors indicate the residue (or species) type: Red-acidic (Asp, Glu), Green-hydrophobic (Ala, Val,Ile, Leu, Tyr, Phe, Trp, Met, Cys, Pro), Purple-basic (Hip, Lys, Arg), Blue-polar (Ser, Thr, Gln, Asn, His, Hie, Hid), Light gray-other (Gly, water) and Darker gray-metal atoms. Interactions with the protein are marked with lines between ligand atoms and protein residues: Solid pink: H-bonds to the protein backbone, Dotted pink: H-bonds to protein side chains, Green: pi-pi stacking interactions, Orange: pi-cation interactions. Ligand atoms exposed to solvent are marked with gray spheres. The protein “pocket” is displayed with a line around the ligand, colored with the color of the nearest protein residue. The gap in the line shows the opening of the pocket. **Figure S2.** 2D interactions of the Kaempferol (A), Astragalin (B), Myricetin (C), Quercetin (D), Isorhamnetol (E), Linoleic acid (F), Oleic acid (G), Stearic acid (H), Palmitic acid (I), ß-sitosterol (J), Luteolin (K), Coumarin (L), α-amyrin (M), and Sesamin (N) with the active site of human serotonin receptor (PDB: 5I6X) for antidepressant activity. **Figure S3.** 2D interactions of the Kaempferol (A), Astragalin (B), Myricetin (C), Quercetin (D), Isorhamnetol (E), Linoleic acid (F), Oleic acid (G), Stearic acid (H), Palmitic acid (I), Luteolin (J), Coumarin (K), and Sesamin (L) with the active site of with COX-1 (PDB: 2OYE) enzyme for anti-nociceptive activity. **Figure S4.** 2D interactions of the Kaempferol (A), Myricetin (B), Quercetin (C), Isorhamnetol (D), Linoleic acid (E), Oleic acid (F), Stearic acid (G), Palmitic acid (H), Luteolin (I), Coumarin (J), and Sesamin (K) with the active site of with COX-2 (PDB: 6COX) enzyme for anti-nociceptive activity. **Figure S5.** 2D interactions of the Kaempferol (A), Astragalin (B), Myricetin (C), Quercetin (D), Isorhamnetol (E), Linoleic acid (F), Oleic acid (G), Stearic acid (H), Palmitic acid (I), Luteolin (J), Coumarin (K), and Sesamin (L) with the active site of M3 muscarinic acetylcholine receptor (PDB ID: 4U14) for antidiarrheal activity. **Figure S6.** 2D interactions of the Kaempferol (A), Myricetin (B), Quercetin (C), Isorhamnetol (D), Linoleic acid (E), Oleic acid (F), Stearic acid (G), Palmitic acid (H), Luteolin (I), Coumarin (J), and Sesamin (K) with the active site of 5-HT3 receptor (PDB ID: 5AIN) for antidiarrheal activity.

## Abbreviations

MECR refers to methanol extract of *Cuscuta reflexa* leaves	
p.o.	per oral;
i.p.	Intraperitoneal;
ANOVA	Analysis of variance;
BW	body weight;
SEM	standard error of mean;
SPSS	statistical package for social science.
ADME/T	Absorption, Distribution, Metabolism, Excretion, and Toxicity;
PASS	Prediction of Activity Spectra for Substances.
