# Supplementary material for: Unveiling Pharmacological Responses and Potential Targets Insights of Identified Bioactive Constituents of Cuscuta reflexa Roxb. Leaves through In Vivo and In Silico Approaches
Source: Pharmaceuticals (Basel). 2020 Mar 20;13(3):50. doi: 10.3390/ph13030050 (PMC7151675; doi:10.3390/ph13030050)
Supplement: Supplementary file 1 [file pharmaceuticals-13-00050-s001.pdf]

Supplementary Materials

Article

Unveiling Pharmacological Responses and Potential Targets Insights of Identified Active Constituents of Cuscuta reflexa leaves through In Vivo and In Silico Approaches

Md. Adnan[1,†], Md Nazim Uddin Chy[2,†], A.T.M Mostafa Kamal[2,*], Md. Riad Chowdhury[2], Md. Shariful Islam[2], Md. Amzad Hossain[2], Md Abu Montakim Tareq[2], Md. Imam Hossain Bhuiyan[2], Md Nasim Uddin[2], Afroza Tahamina[3], Md Obyedul Kalam Azad[1], Young Seok Lim[1], Dong Ha Cho[1,*]

[1] Department of Bio-Health Technology, Kangwon National University, Chuncheon 24341, Republic of Korea; mdadnan1991.pharma@gmail.com (M.A.); azadokalam@gmail.com (M.O.K.A.); potatoschool@kangwon.ac.kr (Y.S.L.);

[2] Department of Pharmacy, International Islamic University Chittagong, Chittagong 4318, Bangladesh, nazim107282@gmail.com (M.N.U.C.); riadchy01@gmail.com (M.R.C.); sharif_305307@yahoo.com (M.S.I.); ctg.amzad.edu@gmail.com (M.A.H.); montakim0.abu@gmail.com (M.A.M.T.); imamhossain037@gmail.com (M.I.H.B.); mdnasimuddin2018@gmail.com (M.N.U.)

[3] Beijing Advanced Innovation Center for Food Nutrition and Human Health, Beijing Technology and Business University 100048; tahaminasompurna@gmail.com (A.T.)

† These authors contributed equally to this work

* Correspondence: chodh@kangwon.ac.kr (D.H.C.); mostafa@pharm.iiuc.ac.bd (A.T.M.M.K.)

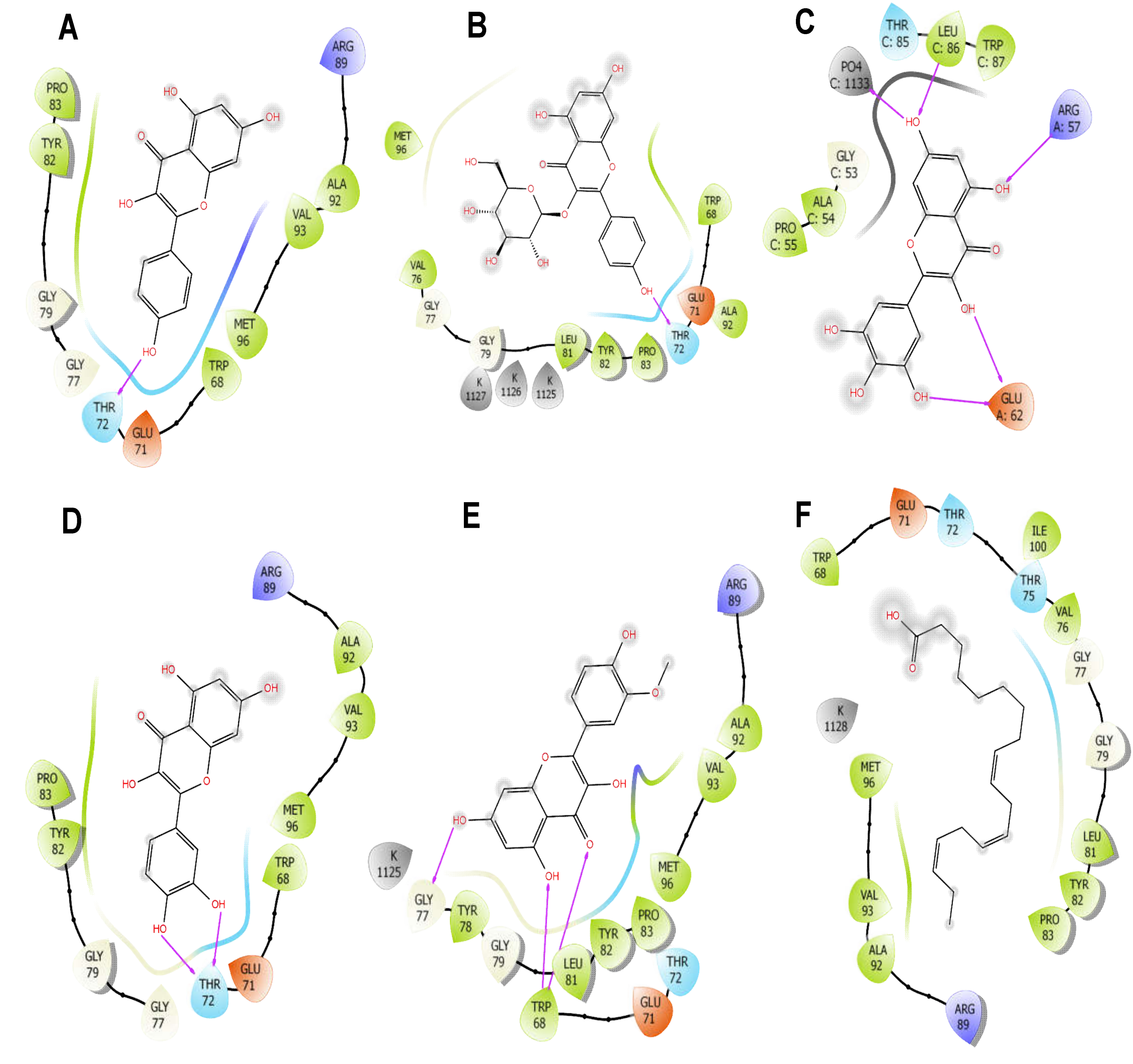

Figure S1. 2D interactions of the Kaempferol (A), Astragalin (B), Myricetin (C), Quercetin (D), Isorhamnetol (E), Linoleic acid (F), Beta sitosterol, Luteolin (G), Coumarin (H), n-Hentriacontane (I), Alpha-amyrin (J), and Sesamin (K)

with the active site of potassium channel (PDB: 4UUJ) receptor for anxiolytic activity. Colors indicate the residue (or species) type: Red-acidic (Asp, Glu), Green-hydrophobic (Ala, Val,Ile, Leu, Tyr, Phe, Trp, Met, Cys, Pro), Purple-basic (Hip, Lys, Arg), Blue-polar (Ser, Thr, Gln, Asn, His, Hie, Hid), Light gray-other (Gly, water) and Darker gray-metal atoms. Interactions with the protein are marked with lines between ligand atoms and protein residues: Solid pink: H-bonds to the protein backbone, Dotted pink: H-bonds to protein side chains, Green: pi-pi stacking interactions, Orange: pi-cation interactions. Ligand atoms exposed to solvent are marked with gray spheres. The protein "pocket" is displayed with a line around the ligand, colored with the color of the nearest protein residue. The gap in the line shows the opening of the pocket.

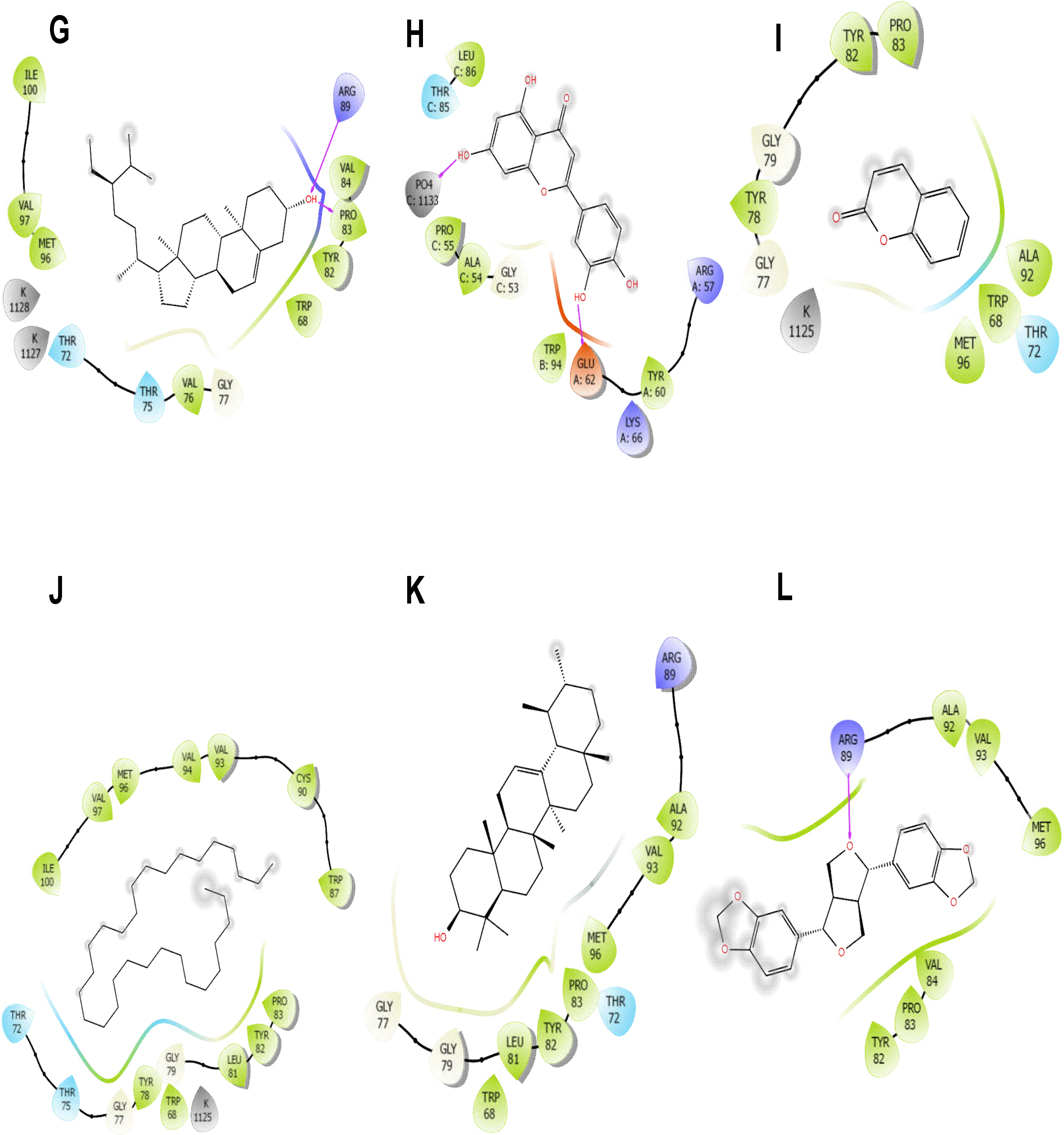

Figure S1 (Continued).

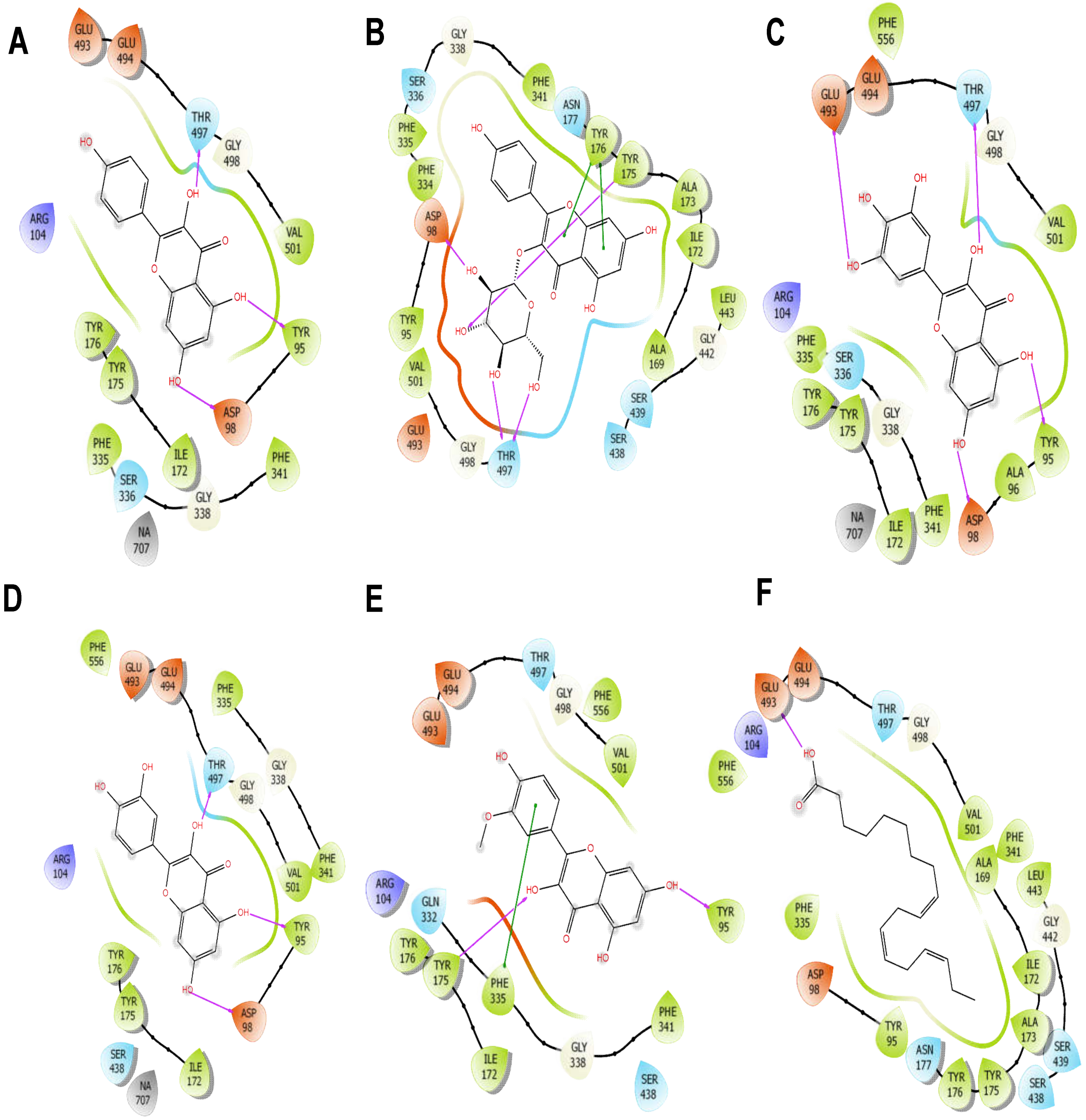

Figure S2. 2D interactions of the Kaempferol (A), Astragalin (B), Myricetin (C), Quercetin (D), Isorhamnetol (E), Linoleic acid (F), Oleic acid (G), Stearic acid (H), Palmitic acid (I), Beta sitosterol (J), Luteolin (K), Coumarin (L), Alpha-amyrin (M), and Sesamin (N) with the active site of human serotonin receptor (PDB: 5I6X) for antidepressant activity

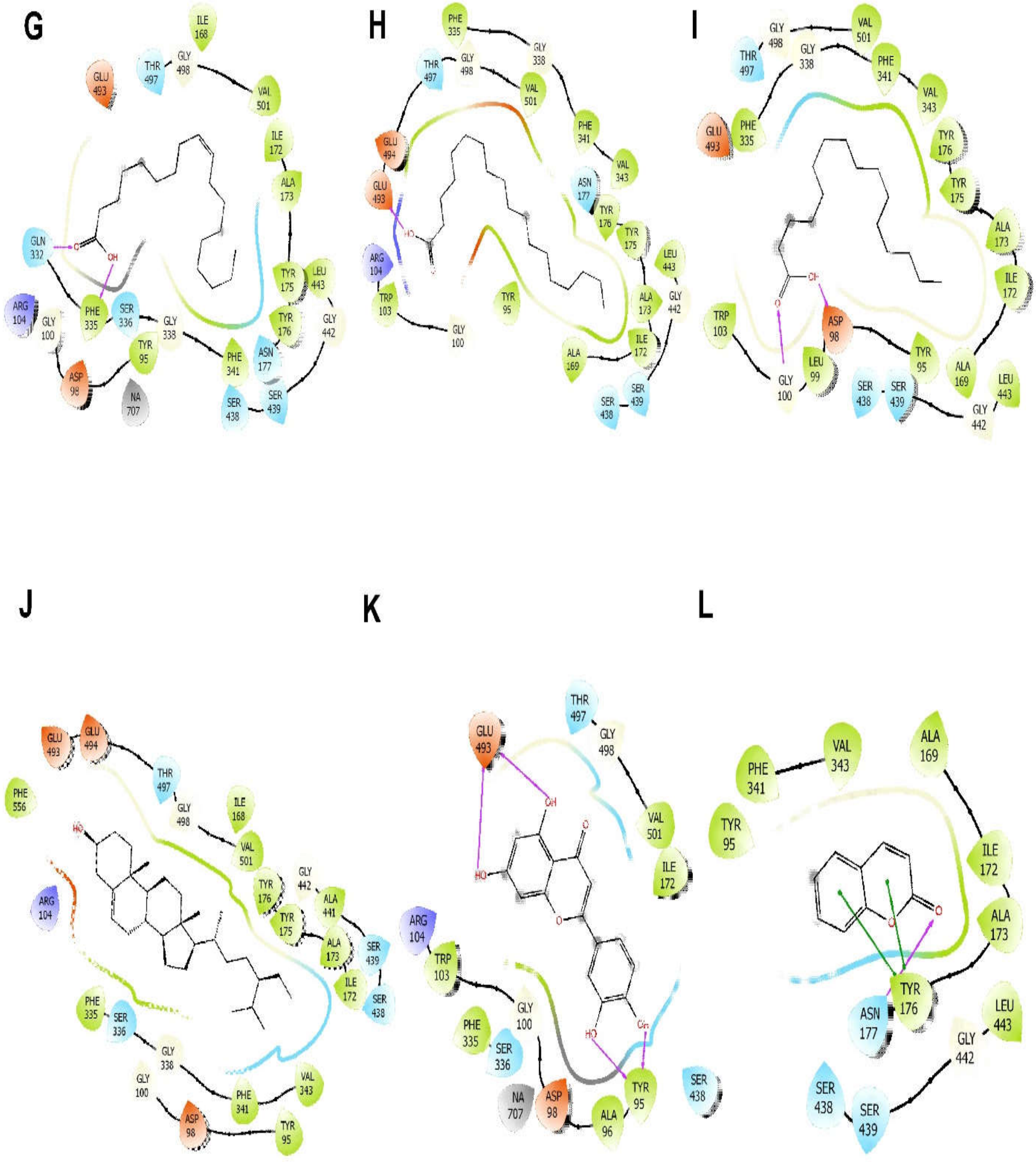
G
ILE 168
THR 497
GLY 498
GLU 493
VAL 501
ILE 172
ALA 173
GLN 332
OH
TYR 175
LEU 443
ARG 104
GLY 100
PHE 335
SER 336
GLY 338
TYR 176
GLY 442
TYR 95
PHE 341
ASN 177
ASP 98
NA 707
SER 438
SER 439
H
PHE 335
GLY 338
THR 497
GLY 498
VAL 501
GLU 494
GLU 493
PHE 341
VAL 343
ASN 177
TYR 176
TYR 175
ARG 104
TRP 103
LEU 443
GLY 442
ALA 173
TYR 95
GLY 100
ILE 172
ALA 169
SER 439
SER 438
I
GLY 498
VAL 501
THR 497
GLY 338
PHE 341
VAL 343
GLU 493
PHE 335
TYR 176
TYR 175
ALA 173
ILE 172
TRP 103
ASP 98
TYR 95
ALA 169
LEU 443
GLY 100
LEU 99
SER 438
SER 439
GLY 442
J
GLU 493
GLU 494
THR 497
GLY 498
ILE 168
PHE 556
VAL 501
HO
GLY 442
TYR 176
ALA 441
ARG 104
TYR 175
ALA 173
SER 439
ILE 172
SER 438
PHE 335
SER 336
GLY 338
GLY 100
VAL 343
PHE 341
ASP 98
TYR 95
K
THR 497
GLY 498
GLU 493
OH
VAL 501
ILE 172
HO
ARG 104
TRP 103
GLY 100
PHE 335
SER 336
HO
NA 707
ASP 98
ALA 96
TYR 95
SER 438
L
VAL 343
ALA 169
PHE 341
TYR 95
ILE 172
ALA 173
TYR 176
ASN 177
LEU 443
GLY 442
SER 438
SER 439

Figure S2 (Continued).

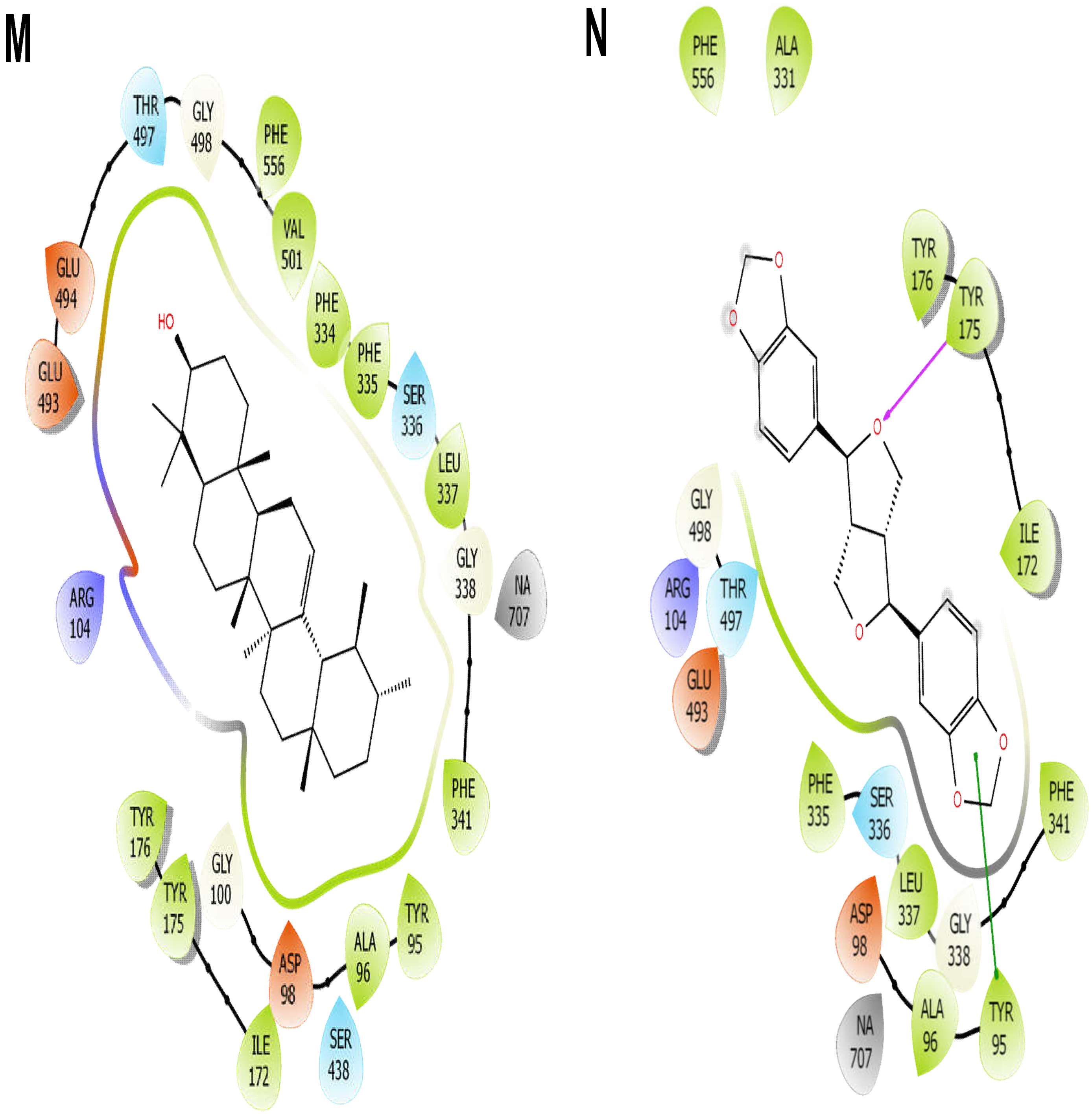

Figure S2 (Continued).

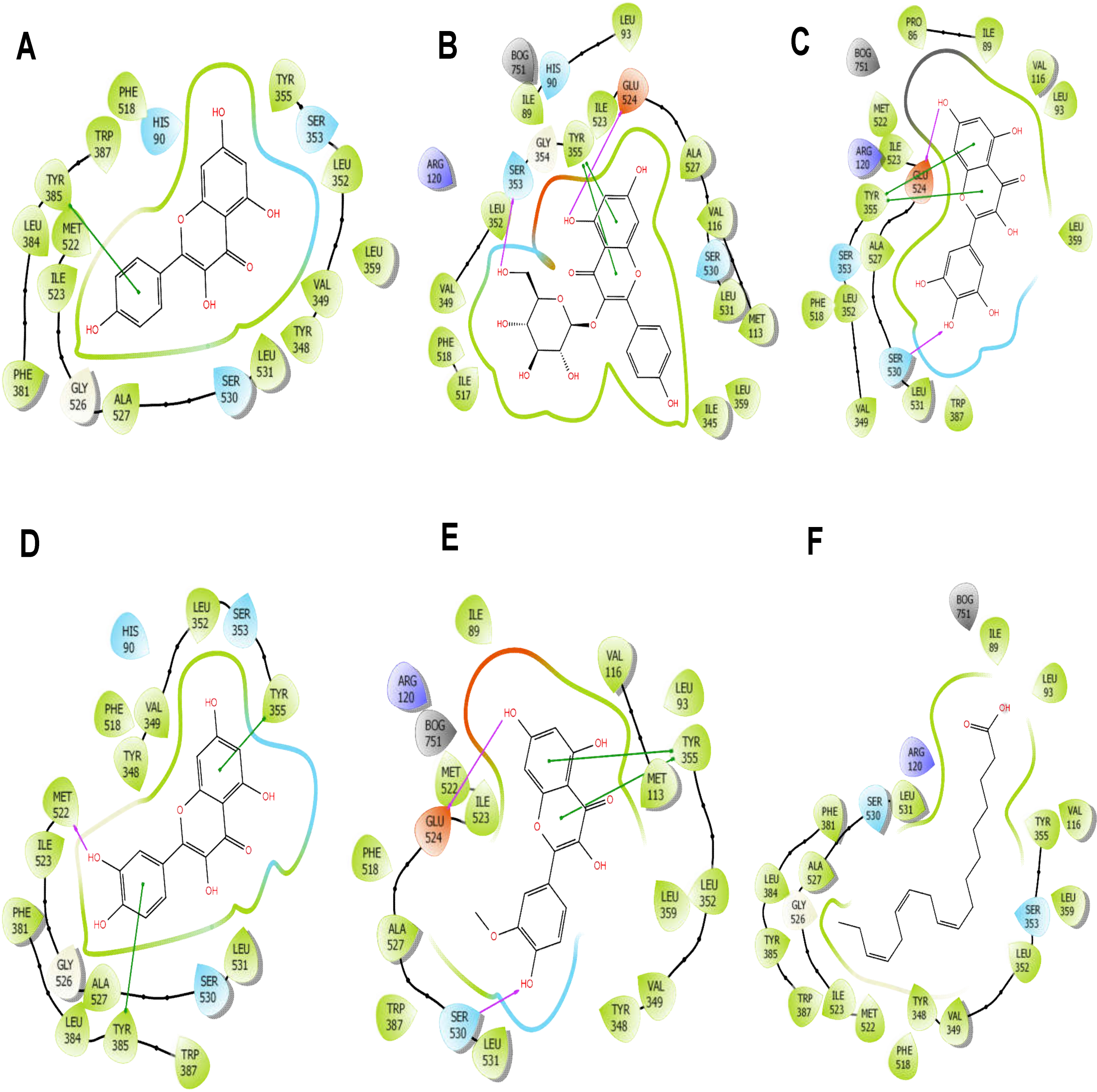

Figure S3. 2D interactions of the Kaempferol (A), Astragalin (B), Myricetin (C), Quercetin (D), Isorhamnetol (E), Linoleic acid (F), Oleic acid (G), Stearic acid (H), Palmitic acid (I), Luteolin (J), Coumarin (K), and Sesamin (L) with the active site of with COX-1 (PDB: 2OYE) enzyme for anti-nociceptive activity.

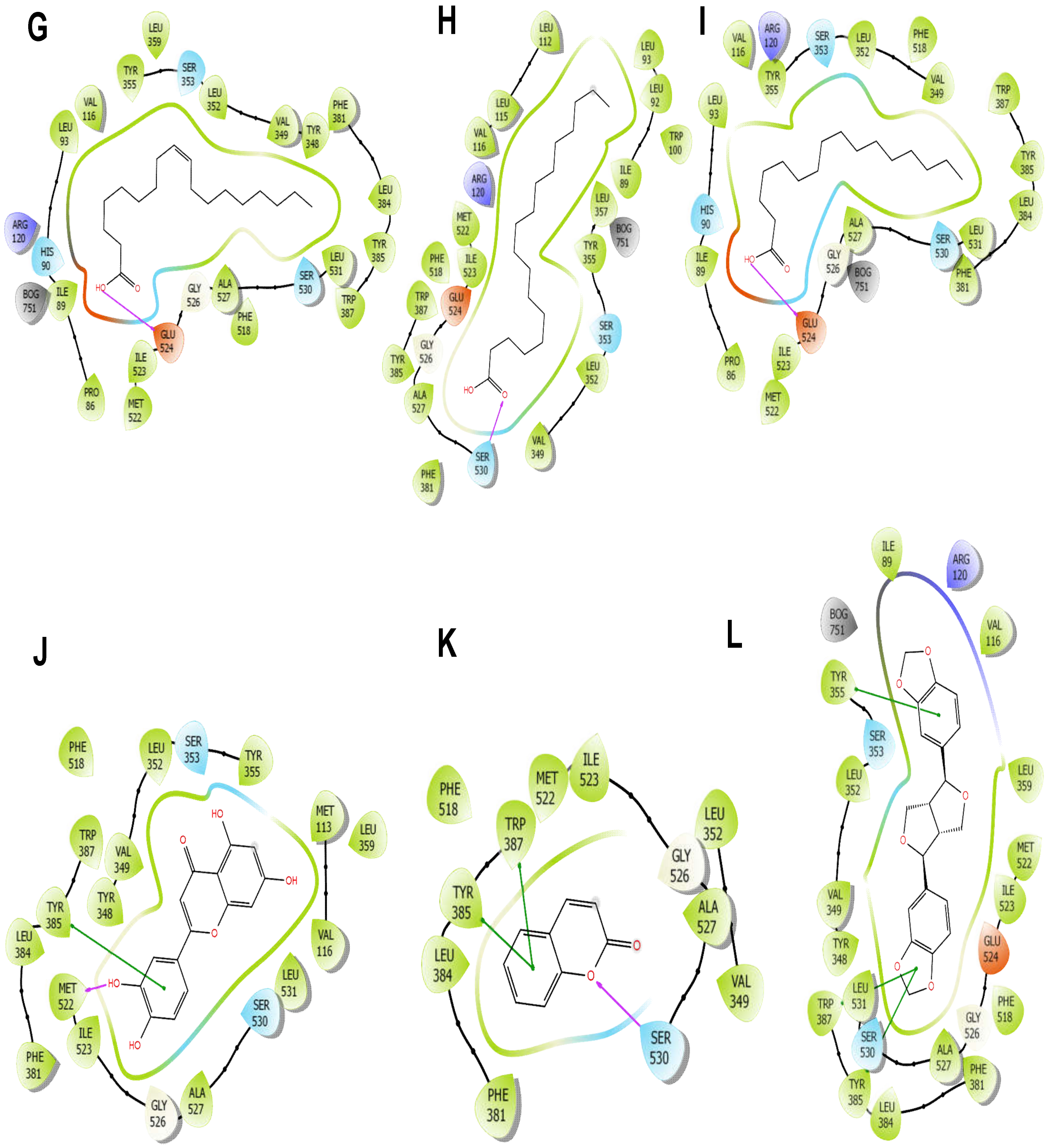

Figure S3 (Continued).

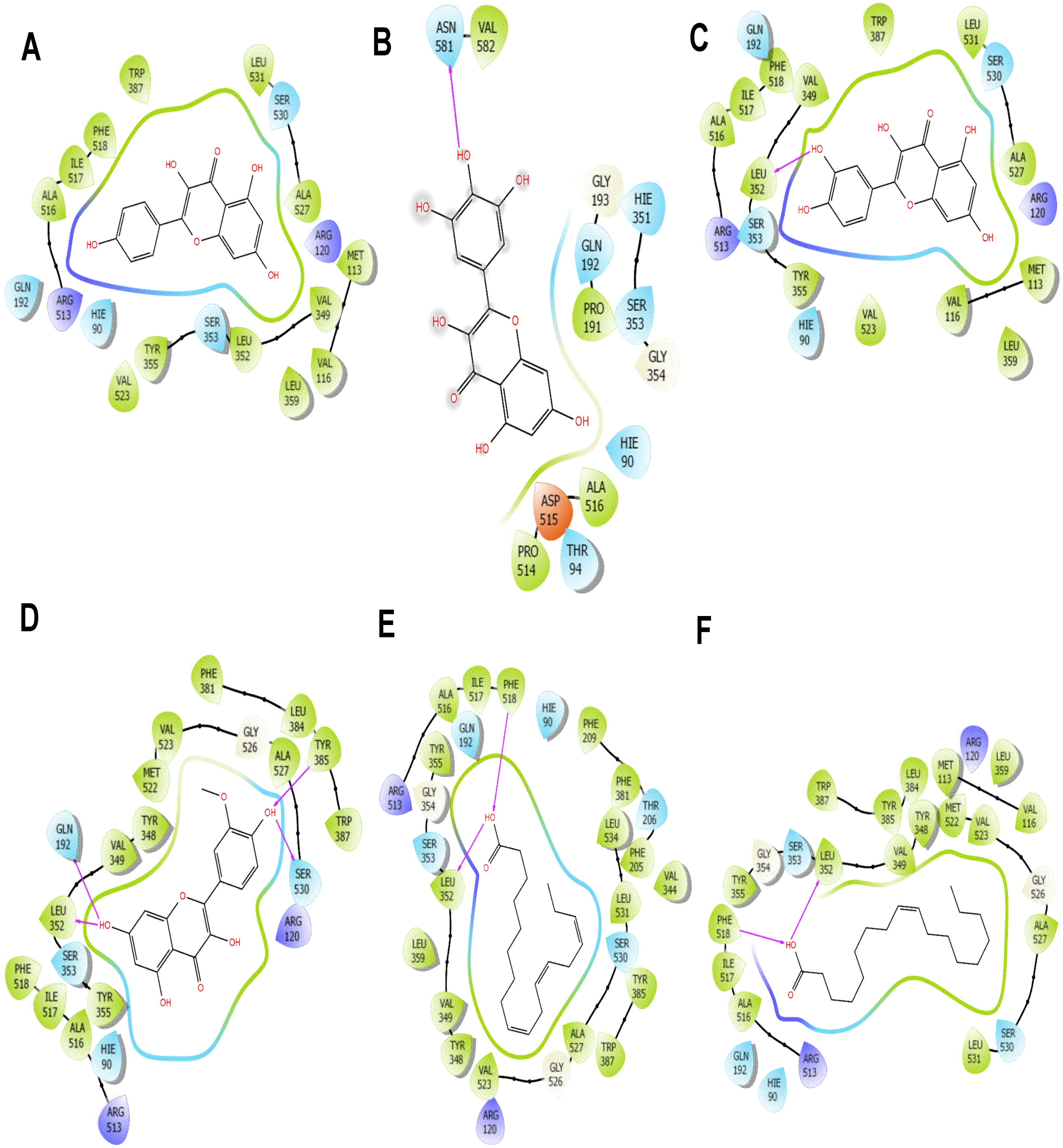

Figure S4. 2D interactions of the Kaempferol (A), Myricetin (B), Quercetin (C), Isorhamnetol (D), Linoleic acid (E), Oleic acid (F), Stearic acid (G), Palmitic acid (H), Luteolin (I), Coumarin (J), and Sesamin (K) with the active site of with COX-2 (PDB: 6COX) enzyme for anti-nociceptive activity

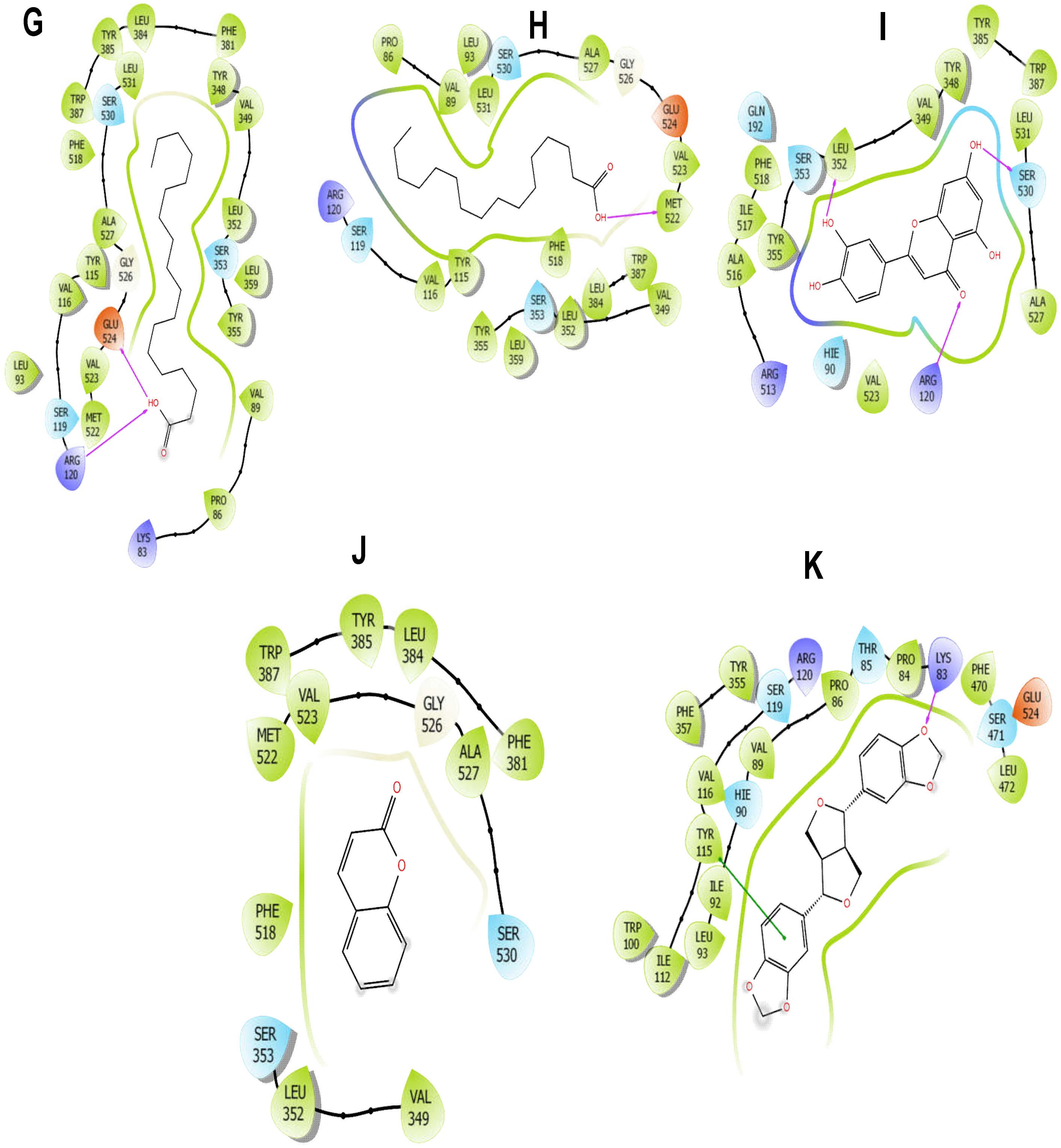

Figure S4 (Continued).

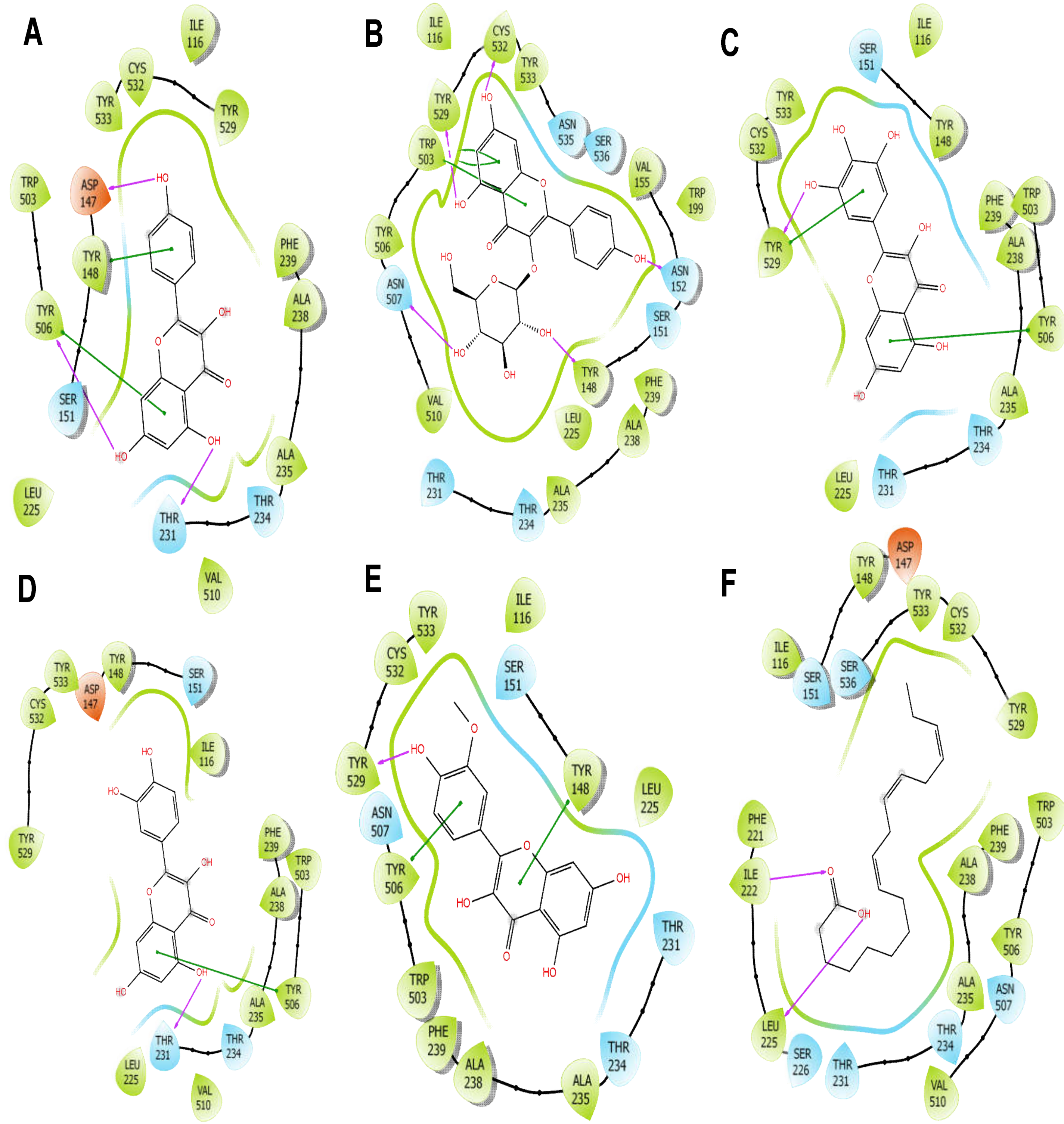

Figure S5. 2D interactions of the Kaempferol (A), Astragalin (B), Myricetin (C), Quercetin (D), Isorhamnetol (E), Linoleic acid (F), Oleic acid (G), Stearic acid (H), Palmitic acid (I), Luteolin (J), Coumarin (K), and Sesamin (L) with the active site of M3 muscarinic acetylcholine receptor (PDB ID: 4U14) for antidiarrheal activity

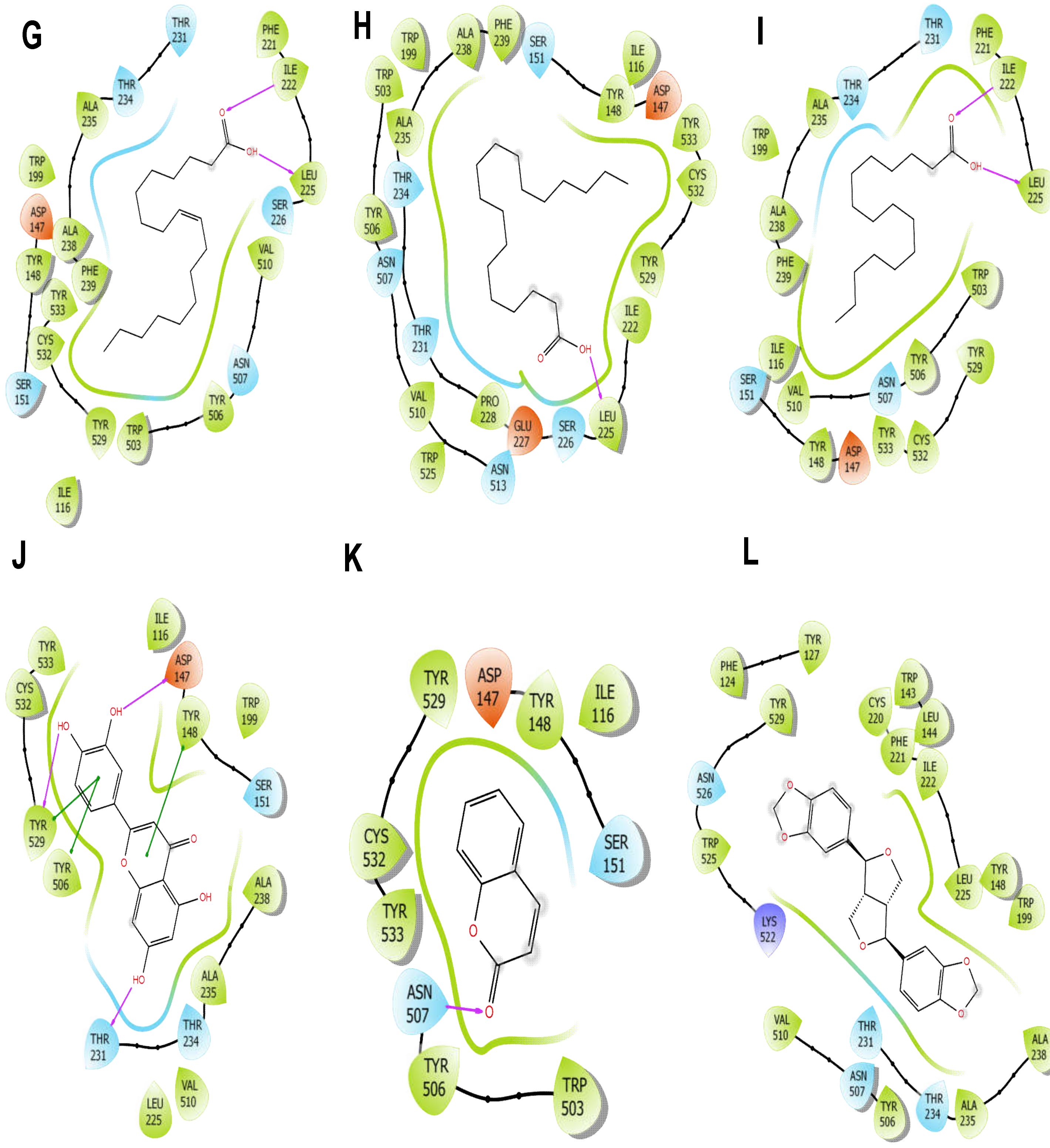

Figure S5 (Continued).

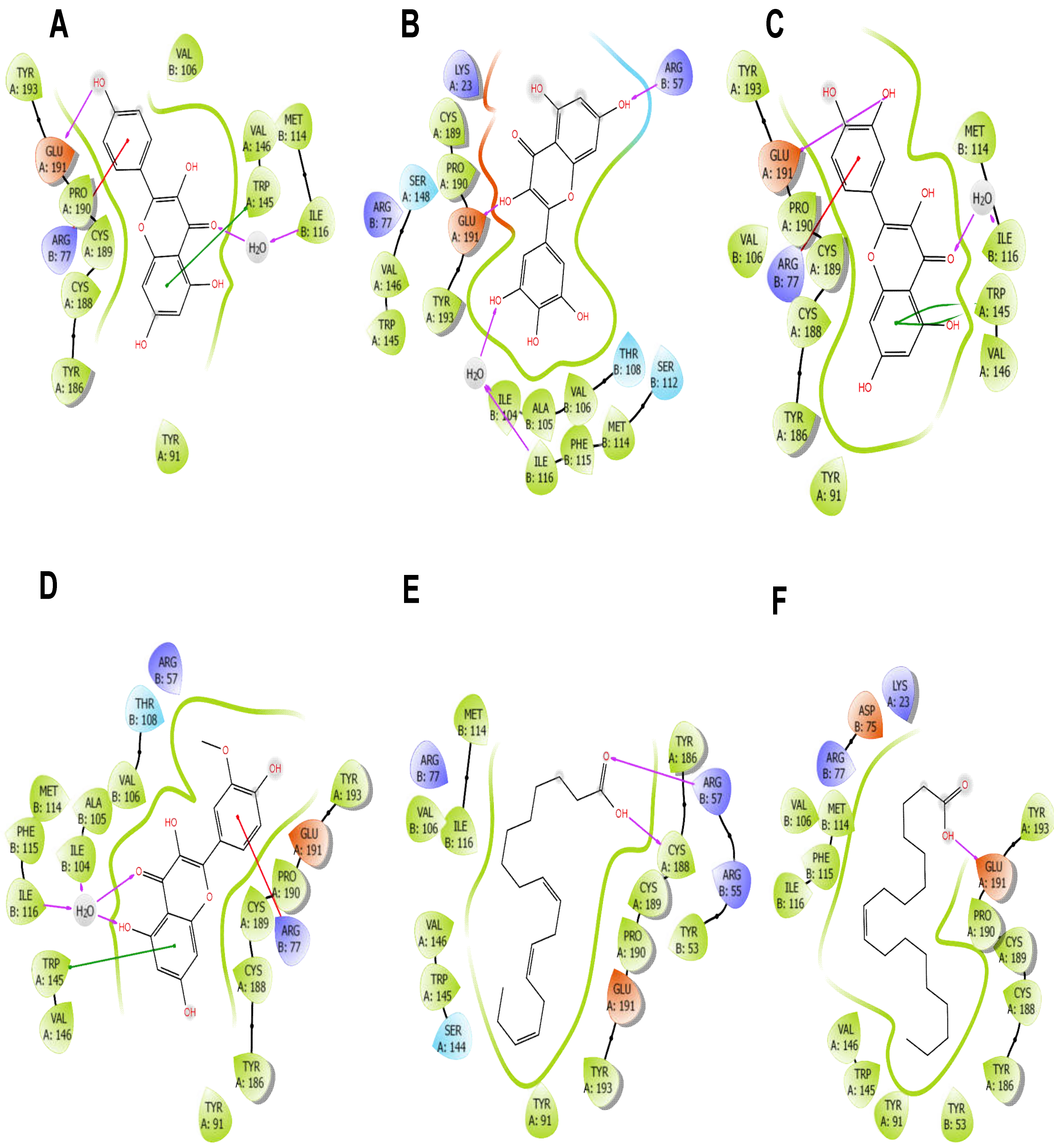

Figure S6. 2D interactions of the Kaempferol (A), Myricetin (B), Quercetin (C), Isorhamnetol (D), Linoleic acid (E), Oleic acid (F), Stearic acid (G), Palmitic acid (H), Luteolin (I), Coumarin (J), and Sesamin (K) with the active site of 5-HT3 receptor (PDB ID: 5AIN) for antidiarrheal activity

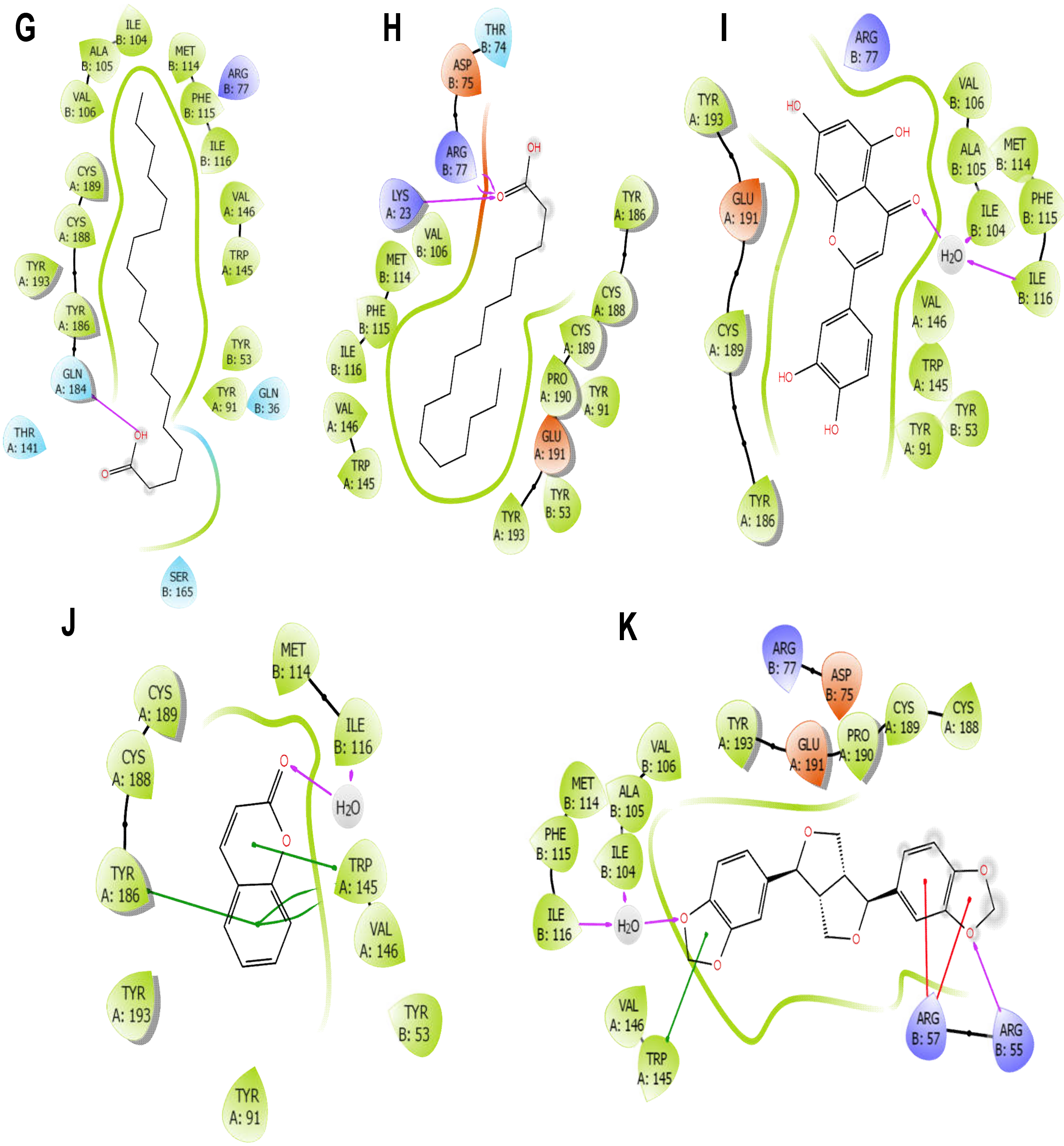

Figure S6 (Continued).
